# Alterations in the Gut Microbiome and Suppression of Histone Deacetylases by Resveratrol Are Associated with Attenuation of Colonic Inflammation and Protection Against Colorectal Cancer

**DOI:** 10.3390/jcm9061796

**Published:** 2020-06-09

**Authors:** Haider Rasheed Alrafas, Philip Brandon Busbee, Kumaraswamy Naidu Chitrala, Mitzi Nagarkatti, Prakash Nagarkatti

**Affiliations:** 1 Department of Pathology, Microbiology, and Immunology, University of South Carolina School of Medicine, Columbia, SC 29209, USA; haider.alrafas@gmail.com (H.R.A.); brandon.busbee@uscmed.sc.edu (P.B.B.); mitzi.nagarkatti@uscmed.sc.edu (M.N.); 2 Fels Institute for Cancer Research and Molecular Biology, Temple University, Philadelphia, PA 19140, USA; tul53710@temple.edu

**Keywords:** colorectal cancer, resveratrol, microbiome, azoxymethane, dextran sodium sulfate, fecal transfer, butyrate, T regulatory cells, T-helper cells, histone deacetylase

## Abstract

Inflammatory bowel disease (IBD) is known to significantly increase the risk for development of colorectal cancer (CRC), suggesting inflammation and cancer development are closely intertwined. Thus, agents that suppress inflammation may prevent the onset of cancer. In the current study, we used resveratrol, an anti-inflammatory stilbenoid, to study the role of microbiota in preventing inflammation-driven CRC. Resveratrol treatment in the azoxymethane (AOM) and dextran sodium sulphate (DSS) CRC murine model caused an increase in anti-inflammatory CD4 + FOXP3 + (Tregs) and CD4 + IL10 + cells, a decrease in proinflammatory Th1 and Th17 cells, and attenuated CRC development. Gut microbial profile studies demonstrated that resveratrol altered the gut microbiome and short chain fatty acid (SCFA), with modest increases in n-butyric acid and a potential butyrate precursor isobutyric acid. Fecal transfer from resveratrol-treated CRC mice and butyrate supplementation resulted in attenuation of disease and suppression of the inflammatory T cell response. Data also revealed both resveratrol and sodium butyrate (BUT) were capable of inhibiting histone deacetylases (HDACs), correlating with Treg induction. Analysis of The Cancer Genome Atlas (TCGA) datasets revealed increased expression of Treg-specific transcription factor FoxP3 or anti-inflammatory IL-10 resulted in an increase in 5-year survival of patients with CRC. These data suggest that alterations in the gut microbiome lead to an anti-inflammatory T cell response, leading to attenuation of inflammation-driven CRC.

## 1. Introduction

Colorectal cancer (CRC), which is characterized by tumor development in the large intestine, ranks as third among cancer incidences and fourth in cancer-related mortalities worldwide [1]. Despite an overall decrease in CRC incidence in the United States among all race and ethnic groups due to standardized screening guidelines [2], there has been a rise in prevalence of this disease among young adult patients, which prompted the American Cancer Society to suggest the recommended age for CRC screening be lowered from 50 to 45 [3]. Even with conventional chemotherapy options, which have major negative side-effects, patients often show chemo-resistance [4]. Moreover, stage IV colon cancer patients have only a 10% chance of a cure. For this reason, the emphasis has been on prevention of CRC development and regular screening to detect and cure such cancer at an early stage. CRC development is also associated with chronic inflammation and high levels of circulating inflammatory biomarkers [5,6]. Recent reports have shown that inflammation induced by certain types of diet and alterations in the microbiome is associated with increased risk of CRC development in men and women [7,8]. By the same token, diet and lifestyles that promote chronic inflammation in the gut are associated with dysregulation in the microbiome and development of colon tumorigenesis [9,10]. Together, such studies suggest that use of preventative agents against colon inflammation, or colitis, could be beneficial in reducing the incidence of CRC.

One such promising preventive measure to suppress chronic inflammation is the use of a resveratrol, a natural phytochemical and aryl hydrocarbon receptor (AhR) ligand found in fruits such as grapes and mulberries, in addition to other consumables such as red wine [11]. Resveratrol, also known as 3,4’,5-trihydroxystilbene, has already been shown by our lab, as well as by others, to possess a myriad of anti-cancer effects, including those related to CRC. This natural compound has been shown to be effective at preventing the proliferation and survival of human CRC cells as well as decreasing CRC disease severity and tumor development in relevant animal CRC models [11,12,13]. Some of the mechanisms by which resveratrol has been shown to prevent colon cancer cell proliferation and invasion metastasis include regulation of key cellular signaling pathways such as NF-Κb-dependent cellular processes [14], PI3K/Akt signaling [15], modulation of histones and sirtuins [16], inhibition of cyclooxygenase-2 (Cox-2) expression [17], and alterations in gene-regulating microRNAs (miR) [18]. Previous reports from our lab have shown that resveratrol is able to alter expression of certain miRs (miR-101b and miR-455) that target inflammatory mediators such as interleukin-6 (IL-6), tumor necrosis factor alpha (TNF-α), and COX-2 in the dextran sodium sulfate (DSS)-induced colitis-associated tumorigenesis Apc (Min/+) mouse model [19]. In addition, in the AOM/DSS CRC model, we have shown that resveratrol downregulates inflammatory stress markers such as p53 to modulate the T cell response [20]. However, while the regulation of these host-derived cellular mechanisms plays an important role in resveratrol-mediated CRC treatment, recent research has shown the gut microbiome is also a key player in both CRC disease development and progression [21]. Thus, whether the ability of resveratrol to suppress CRC is related to its action on gut microbiota remains a possibility that needs to be explored.

The gut microbiome, a diverse ecosystem consisting of gut commensals including bacteria and fungi, has been shown to have a great impact on human health and disease, particularly in CRC [22,23,24]. For example, patients diagnosed with CRC were found to have distinct microbiome profiles compared to healthy controls, and this microbial signature was found to be altered after treatment with probiotics [25]. Interestingly, after oral administration of antibiotics to deplete the gut microbiome, tumor burden was decreased in a CRC murine model, but this effect was negated in Rag-deficient mice that lacked mature T cells and B cells [26]. This study highlights the importance of the interplay between the host immune defense and the microbiome. Resveratrol has been shown to modulate the host immune response to promote anti-inflammation as previously discussed and has been shown to alter the microbiome in other disease models [27,28,29,30]. For example, resveratrol has been shown to increase the abundances of bacteria known to produce short-chain fatty acid (SCFA) butyrate, a microbial byproduct which exerts anti-inflammatory effects [31,32]. In addition, activation of AhR by ligands such as resveratrol has previously been shown to alter the gut microbial composition by regulating host intestinal cell responses, such as the release of sensing molecules like interleukin-22 (IL-22) or direct effects such as the release of anti-microbial peptides (AMPs) [33,34,35]. In fact, we recently published a report showing how another AhR ligand was able to alter colonic IL-22 levels to prevent colitis-associated microbial dysbiosis [36]. However, there are currently no reports determining if resveratrol-mediated alterations in the gut microbiome can influence the immune response to protect against CRC development. The current study was undertaken to address this issue, and we show that in the AOM/DSS CRC murine model, resveratrol was able to alter the gut microbiome profile and SCFA microbial-production, mainly by altering n-butyric acid and isobutyric (i-butyric) acid, also known as 2-iodobutanoic acid or 2-methylpropanoic acid. These alterations promoted an anti-inflammatory T cell response (Treg and CD4 + IL-10), which decreased disease severity and tumor development in the colon. Additionally, fecal transfer from resveratrol-treated mice and butyrate supplementation experiments showed that modulation of gut microbiota and suppression of histone deacetylases (HDACs) were key mechanisms through which resveratrol was able to regulate the immune response and prevent CRC development. Lastly, we showed that in the human CRC population, increased expression of Treg-associated genes (FoxP3 and IL-10) correlates with increased survival rates, thereby providing additional proof of the role of anti-inflammatory environment in CRC suppression.

## 2. Experimental Section

Animals. Female C57BL/6 mice (aged 6–8 weeks) were purchased from Jackson Laboratories (Bar Harbor, ME, USA), and all mice were housed at the AAALAC-accredited animal facility at the University of South Carolina, School of Medicine (Columbia, SC, USA). All procedures were performed according to NIH guidelines under protocols approved by the University of South Carolina Institutional Animal Care and Use Committee (IACUC).

Induction of AOM/DSS CRC in mice and treatment(s). To test the efficacy of treatment in AOM/DSS-induced CRC model, AOM was purchased from Sigma-Aldrich (St. Louis, MO, USA) and administered one time via intraperitoneal (i.p.) injection into C57BL/6 mice at a dose of 10 mg/kg at day zero, followed by three cycles of 2% DSS (Chem-Impex International, Wood Dale, IL, USA) as previously described [20]. For treatment groups, resveratrol (Sigma-Aldrich, St. Louis, MO, USA) was administered by oral gavage at 100 mg/kg suspended in 100 µl of water, as previously reported [37]. The regimen for resveratrol consisted of administering this compound 24 h prior to the injection of AOM, followed by daily treatment throughout the duration of the experiment (10 weeks). Control groups consisted of naïve mice receiving either normal water or 100 mg/kg resveratrol. For butyrate supplementation experiments, sodium butyrate (BUT) from Sigma-Aldrich was given to mice at 200 mg/kg dissolved in water using the same regimen (days and controls) as resveratrol for 10 weeks.

Procedures for evaluating CRC disease severity. During AOM/DSS induced CRC, mice were weighed daily after AOM injection. Animals were euthanized at the experimental endpoint (10 weeks after AOM injection) for further evaluation of clinical signs to include counting the number and size of tumors in the colon highlighted by 1% Alcian blue dye and measured by a ruler or digital caliper. Colonoscopies were performed weekly to experimental groups using a Karl Storz (Tuttlingen, Germany) Tele Pack Vet X LED endoscope and scored the following way: 0 = normal colon, 1 = presence of blood and tissue sloughing, 2 = presence of 1–2 colonic polyps, 3 = 3−5 colonic polyps present, 4 = 5−10 colonic polyps present, 5 = <10 polyps present in the colon. Colon, mesenteric lymph node (MLN), spleen, and blood were collected from euthanized mice for further evaluation. Colons were cleaned by saline wash and sectioned for histological analysis. Colon sections (tumor and normal adjacent tissue) were fixed with 4% paraformaldehyde and embedded in paraffin, cut into 5 µm sections, deparaffinized in xylene, serially diluted in decreasing concentrations of ethanol, and stained with hematoxylin-eosin (H&E) and Periodic Acid Schiff (PAS) staining kits (Sigma-Aldrich, St. Louis, MO, USA). Images of stained sections were taken using a Biotek (Winooski, VT, USA) Cytation 5 with digital wide field microscopy capabilities.

Cellular phenotyping by flow cytometry. Cells from MLN, spleen, and blood were isolated from experimental groups and lysed with RBC lysis buffer (Sigma-Aldrich, St. Louis, MO, USA) before being filtered and stained with appropriate antibodies for cellular phenotyping using flow cytometry. All cells were pre-blocked with Fc receptor, washed with FACS staining buffer (PBS with 2% fetal bovine serum), and stained with commercially-available antibodies (Biolegend, San Diego, CA, USA) as follows: FITC-anti-CD3, PE-anti-CD8, and PE-CY7-anti-CD4 to identify T cells and FITC-anti-Gr1 and PE-anti-CD11b to identify myeloid-derived suppressor cells (MDSCs). For phenotyping of T cell subsets, intracellular (Intracellular Staining Permeabilization Wash Buffer) and intranuclear (True-Nuclear Transcription Factor Buffer Set) staining kits (Biolegend, San Diego, CA, USA) were used by way of manufactures instructions. Permeabilized cells were stained with PE-Cy7-anti-CD4, PE-anti-Foxp3, FITC-anti-IL10, PE-anti-IFNγ, and/or FITC-anti-IL17 (Biolegend, San Diego, CA, USA). Flow cytometry data were analyzed using a CXP FC500 flow cytometry (Beckman Coulter, Brea, CA, USA).

In vitro treatment of activated splenocytes with resveratrol or BUT. For in vitro experiments treated with resveratrol or BUT, whole splenocytes were excised from 8–10 week old C57BL/6 mice and single cell suspensions were cultured in anti-CD3-coated (0.5µg/mL) 96-well plates at 1 × 10^6^ cells/mL density in complete RPMI media for 24 h at 37 °C, 5% CO_2_. Cultured cells were then activated with soluble anti-CD28 (2 µg/mL) in the presence or absence of resveratrol (5, 10, or 25 µM) or BUT (1 mM, 5 mM, or 10 mM) for 24 h (37 °C, 5% CO_2_). In vitro doses of resveratrol were based on previous reports from our lab [38]. The range of doses for BUT was determined using information gathered from other publications [39,40].

16S rRNA gut microbiota profiling and phylogenetic investigation of communities by reconstruction of Unobserved States (PiCRUSt). 16S rRNA gut microbial profiling and SCFA quantification were done as previously described in our lab [41]. Briefly, colonic contents were collected immediately after euthanasia and gathered in 2 mL Eppendorf tubes while under anaerobic conditions and stored at −80 °C for downstream analysis purposes. For 16S rRNA sequencing, genomic DNA was extracted from 100 mg of colonic flush contents by using the QIAamp DNA Stool Mini Kit (Qiagen, Valencia, CA, USA) according to instructions from the manufacturer. DNA libraries were prepped by amplification of the 16S rRNA V3-V4 hypervariable region with added Illumina adapter overhang nucleotide sequences and sequencing with Illumina (San Diego, CA, USA) MiSeq platform. Sequenced reads were than analyzed using Nephele (https://nephele.niaid.nih.gov), an open-source analysis tool provided by the National Institute of Allergy and Infectious Diseases (NIAID) Office of Cyber Infrastructure and Computational Biology (OCICB) in Bethesda, MD [42]. For microbial profiling, QIIME FASTQ paired end with chimera removal, open reference, and SILVA rRNA database project (Silva_99) options were used. For PiCRUSt data, a closed reference against the Greengenes database (Greengene_99) option was used. Operational taxonomic unit (OTU) tables generated from Nephele were further subjected to Linear Discrimination Analysis Effect Size (LEfSe) provided by the Huttenhower group (https://huttenhower.sph.harvard.edu/galaxy/) [43].

SCFA Analysis. For quantification of SCFAs present in colonic flushes, HP 5890 gas chromatograph configured with flame-ionization detectors (GC-FID) was performed as previously described [41,44]. SCFA detection by GC-FID was quantified using Varian MS Workstation (version 6.9.2.) software, and concentrations were calculated by using standards for the detectable SCFAs.

Fecal transfer (FT) experiments. For FT experiments, colonic contents were collected immediately after euthanasia, gathered in 2 mL Eppendorf tubes while under anaerobic conditions, and placed in 30% glycerol solution prior to inoculation into recipient mice. Before FT inoculation, recipient mice were treated with 1 g/L of streptomycin and penicillin dissolved in water and orally gavaged at 100 µL total volume daily for four weeks to deplete endogenous gut microbiota. Depletion of microbiota was validated by PCR analysis using the universal 16S rRNA Eubacteria primer. AOM/DSS CRC induction was performed as previously described in recipient mice. Forty-eight h after the last treatment with the antibiotic cocktail, recipient mice were given fecal material collected from the following groups: Naïve, Resveratrol-treated only, AOM, and AOM + Resveratrol. FT treatments were given via oral gavage on even days for a total of 35 days. Body weight and other clinical parameters described previously were also performed for the FT experiments to include weekly colonoscopies, 1% Alcian blue staining for quantification of colonic tumors, colon histology (H&E and PAS stains), and flow cytometry for analysis of T cell subsets.

Quantitative PCR (qPCR) for bacterial species validation and HDAC expression. For validation of bacteria identified by 16S rRNA analysis, qPCR was used with primers designed to identify the 16s rRNA subunit of significantly altered bacterial species. DNA was extracted from colonic samples using the QIAamp DNA Stool Mini Kit (Qiagen, Venlo, The Netherlands) as previously described. For HDAC expression data, RNA was extracted from single cell suspensions of MLN (in vivo) or cultured splenocytes (in vitro) using RNeasy Mini kits (Qiagen, Venlo, The Netherlands) followed by conversion to cDNA using iScript synthesis kit (Bio-Rad, Hercules, CA, USA). PCR amplification was performed using QuantiFast (bacteria) or QuantiTech (HDAC) SYBR Green PCR kits from Qiagen, and reactions were performed on a CFX96 qPCR system from Bio-Rad (Hercules, CA, USA). Primers were designed by Integrated DNA Technologies (Coralville, IA, USA). Sequences for all primers are included in Table 1.

Correlation of gene expression with survival in CRC patient data sets. The correlation of gene expression pattern with survival of human patients with CRC was performed using the TCGA datasets for colorectal cancer from The Cancer Genome Atlas maintained at TCGA (https://cancergenome.nih.gov/). TCGA examines the genome-wide expression, copy number variations, methylation status, and mutations in an immense number of samples with a primary advantage such as (i) each patient sample is accompanied with a comprehensive clinico-pathological data, (ii) a huge portion of the samples with integrated molecular profiles, (iii) number of matched normals for tumor samples, and (iv) generation of data using latest and widely measured standard molecular profiling technologies [45]. Survival analysis for the TCGA datasets were performed using the Kaplan–Meier survival curves, which is defined as the probability of survival in a given length of time while considering time in many small intervals [46]. It mainly involves the calculation of the probability of occurrence of an event at a certain point of time. The TCGA data was obtained through access to the public domain and is therefore exempt from University of South Carolina Institutional Review Board (IRB) approval.

Statistical analysis. GraphPad Prism software (San Diego, CA, USA) was used for all statistical analysis unless otherwise stated. Experiments were repeated at least three times to confirm reproducibility. For statistical differences, one-way ANOVA and Tukey’s post-hoc comparison test was used unless otherwise noted in the text or figure legends. For analysis measuring the degree of association between sequenced OTU abundances of bacterial species with other variables (e.g., clinical parameters, immune responses, HDAC expression), Spearman rank correlations were performed in GraphPad Prism. Significance was determined to have a *p*-value of ≤0.05 (*), 0.01 (**), 0.005 (***), or 0.001 (****).

Data Availability. The authors declare that all the data supporting the findings of this study are provided within the manuscript, additional information file, and provided links. Raw sequencing data (FASTq files) were uploaded to the NCBI Sequence Read Archive (SRA). For resveratrol treatment experiments, raw reads from experimental groups are under accession number PRJNA528605. For sodium butyrate supplementation experiments, raw reads from experimental groups are under accession number PRJNA528631.

## 3. Results

### 3.1. Resveratrol Attenuates AOM-Induced CRC by Preventing Early Onset of Inflammation and Decreasing Tumor Burden

**Figure 1:**
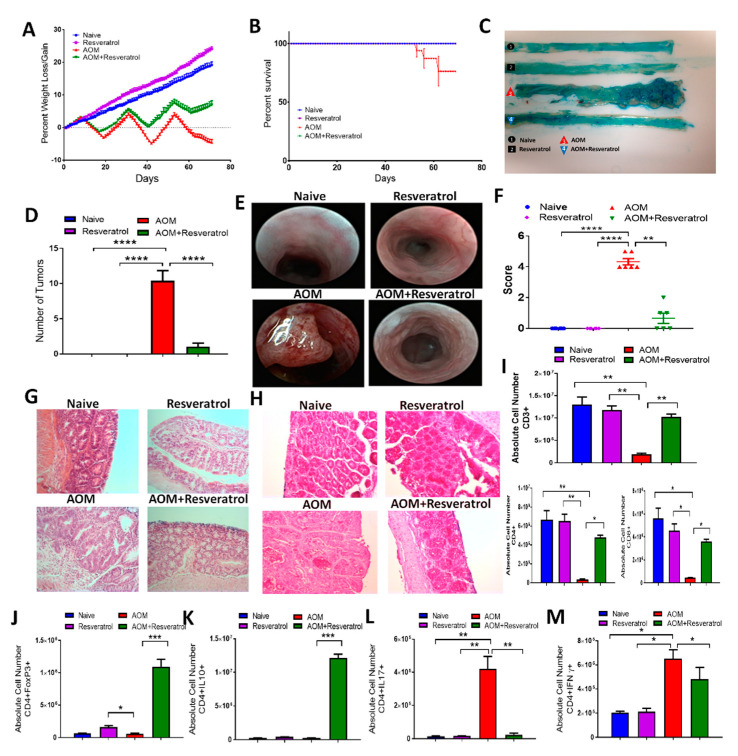
Treatment with resveratrol reduces clinical symptoms and alters T cell phenotype in azoxymethane (AOM)-induced colorectal cancer (CRC) model. C57BL/6 mice were injected intraperitoneal with 10 mg of AOM followed by 3 cycles of 2% DSS, to induce CRC. Experimental groups consisted of: Naïve (*n* = 6), Resveratrol (*n* = 6), AOM (*n* = 6), and AOM+Resveratrol (*n* = 6). Clinical parameters consisted of percent weight loss (**A**) and survival (**B**). (**C**) Representative colons stained with 1% Alcian blue. (**D**) Bar graph depicting number of tumors counted in each experimental group. (**E**) Representative colonoscopic images from experimental groups. (**F**) Bar graph depicting scores after examination of tumor polyps detected during colonoscopies. (**G**) Representative colon sections stained with H&E; scale bar = 100 µM at 40x objective. (**H**) Representative colon sections with PAS staining; scale bar = 100 µM at 40x objective. (**I**) Bar graphs depicting absolute cell numbers in mesenteric lymph node (MLN) for all T cells (CD3+), T helper (CD3+CD4+), and cytotoxic (CD3+CD8+) T cells. (**J**–**M**) Bar graphs depicting absolute cell numbers in MLN for Tregs (**J**), Th cells producing IL-10 (**K**), Th17 (**L**), and Th1 (**M**) cells. Significance (*p*-value: * *p* < 0.05, ** *p* < 0.01, *** *p* < 0.005, **** *p* < 0.001) was determined by using one-way ANOVA and post-hoc Tukey’s test for bar/dot graphs, Mann–Whitney test for weight data, and log rank test for survival curve. Data are representative of at least 3 independent experiments.

To study the effects of resveratrol on CRC in the context of studying host immune response and microbiome, we used the well-characterized AOM/DSS CRC murine model. For these studies, experimental groups consisted of naïve mice (Naïve), naïve mice treated with only resveratrol (Resveratrol), AOM/DSS disease mice treated with AOM and DSS, and AOM/DSS disease mice treated with resveratrol (AOM + Reservatrol). Inducing CRC by AOM resulted in a significant decrease in body weight (~20%) compared to controls (naïve or resveratrol-treated only), but treatment of CRC mice with resveratrol reduced this disease-associated weight loss and resulted in ~8% weight gain by the end of the study (Figure 1A). In addition, the administration of AOM resulted in decreased survival of mice (~75%) by the end of the study, whereas CRC mice treated with resveratrol resulted in 100% survival (Figure 1B). Resveratrol treatment also was able to reduce tumor burden in AOM-induced CRC mice as assessed during the experimental endpoint (10 weeks), as AOM-treated mice developed at least 10 or more tumor polyps along the colon, whereas AOM + Reservatrol mice had little to no tumors polyps present (Figure 1C,D). In order to monitor the first signs of inflammation and tumor development during disease and treatment, colonoscopies were performed weekly among experimental groups. In AOM mice, inflammation development, which was characterized by the presence of bloody lesions and tissue sloughing along the colon, developed around week 3 of the disease model, but AOM + Resveratrol mice maintained more normal appearing colons (Figure S1). The presence of tumors was seen in AOM mice by week 5 and continued developing to week 9, but CRC mice treated with resveratrol showed a marked decrease in colonic tumor development (Figure 1E,F; Figure S1). Colon histology reinforced these observations as AOM colons showed loss of normal mucosal architecture and abnormal tissue growth with standard H&E staining, which was not apparent in AOM + Resveratrol colon sections that more closely resembled controls (Figure 1G). PAS staining on fixed colon sections was also performed as a way to access mucin production and goblet cell formation [47]. Mice challenged with AOM showed a high reduction in the number of goblet cells and presence of mucus compared to controls, but these observations were greatly reversed in colon sections excised from AOM + Resveratrol groups (Figure 1H). Collectively, these data demonstrated that resveratrol treatment attenuated tumor development in the AOM CRC model, perhaps by way of preventing early signs of inflammation caused by multiple cycles of DSS, as shown in week 3 of the colonoscopy images.

### 3.2. Resveratrol Treatment Reduces Inflammatory T Cell Subsets While Increasing Anti-Inflammatory T Cells in AOM-Induced CRC

In order to examine immune cell alterations during disease and treatment, cells were isolated from the MLN, spleen, and blood of all experimental groups and phenotyped using flow cytometry (Figures S2–S5). In the MLN, expression of T cell marker (CD3+), along with T helper (CD3+CD4+) and cytotoxic T cell (CD3+CD8+), were significantly decreased in AOM mice compared to controls, and restoration of these T cell phenotypes occurred in the AOM+Resveratrol groups (Figure 1I). These data suggested that activated T cells in AOM group were leaving MLN and going to the colon while resveratrol reversed this. Similar observations were seen in both the spleen (Figure S3) and the blood (Figure S4). Going further in phenotyping the CD4+ subsets, intracellular/intranuclear staining was performed to identify the effect of resveratrol inflammatory (IFNγ- and IL17-producing) cells) and to anti-inflammatory (FOXP3 + Tregs and IL10-producing) CD4 + T cell populations. The data collected from the MLN showed that there was a significant increase in both anti-inflammatory CD4 + FOXP3 + (Figure 1J) and CD4 + IL10 + (Figure 1K) cells population in AOM mice treated with resveratrol when compared with AOM disease mice. However, proinflammatory T cell subsets, such as Th17 (Figure 1L) and Th1 (CD4 + IFNγ+) (Figure 1M) were significantly higher in AOM mice compared to the controls, but treatment with resveratrol was able to effectively reduce these inflammatory T cell phenotypes. This shift in the proinflammatory to anti-inflammatory T cell subsets after resveratrol treatment was also observed in the spleen (Figure S3). Lastly, as MDSCs are known to increase in the CRC human population and are thought to be a potential immunotherapy target [48], data collected from the spleen and blood revealed that MDSCs were significantly increased in the AOM disease state but were effectively reduced by treatment with resveratrol (Figure S5). Together, these data suggested that resveratrol promoted an anti-inflammatory T cell response in the AOM CRC model.

### 3.3. Alterations in Gut Microbiota and SCFA Composition in AOM-DSS Colorectal Induced Mice Treated With Resveratrol

**Figure 2:**
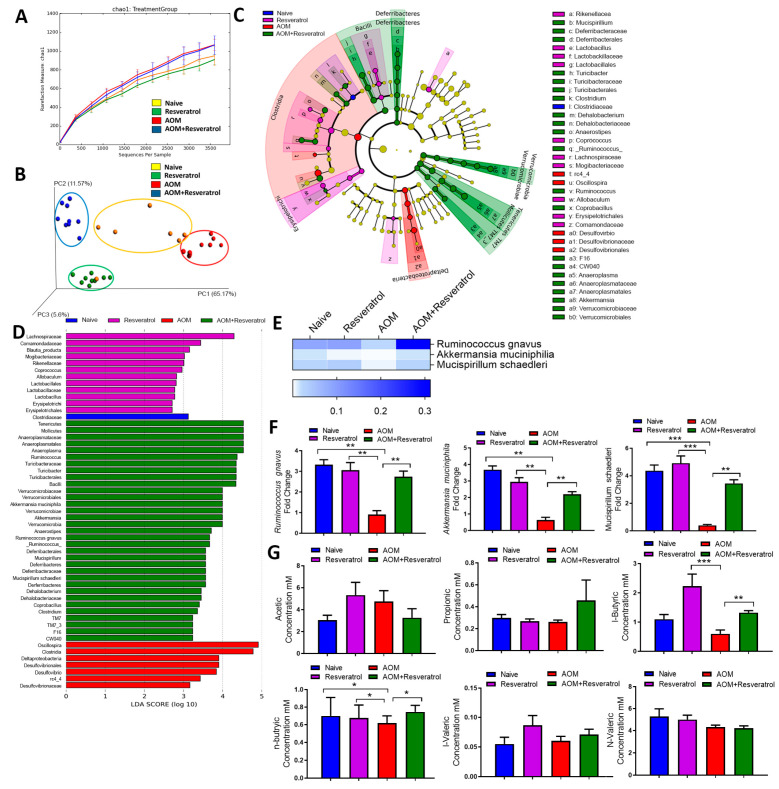
16S rRNA sequencing analysis during AOM-induced CRC treated with resveratrol. The study was designed as described in Figure 1 legend. Gut microbiome samples were collected from experimental groups by performing colonic flushes in experimental groups, which were the following: Naïve (*n* = 7), Resveratrol (*n* = 9), AOM (*n* = 10), and AOM+Resveratrol (*n* = 9). Nephele analysis (nephele.niaid.nih.gov) was used to generate charts for chao1 alpha diversity (**A**) and PCA beta diversity (**B**). LeFSe analysis of the Nephele OTU output files generated the cladogram (**C**) and LDA score bar graph (**D**). (**E**) Heatmap and legend depicting mean OTU percent abundances of significantly altered species *Ruminococcus gnavus*, *Akkermansia muciniphila*, and *Mucispirillum schaedleri*. Rows represent bacterial species and columns represent the mean OTU percentages of experimental groups with SEM. (**F**) PCR validation of *Ruminococcus gnavus*, *Akkermansia muciniphila*, and *Mucispirillum schaedleri.* (**G**) Bar graphs representing concentration of SCFAs acetic acid, propionic acid, i-butyric acid, n-butyric acid, i-valeric acid, and n-valeric acid. Significance (*p*-value: * *p* < 0.05, ** *p* < 0.01, *** *p* < 0.005) was determined by using one-way ANOVA followed by Tukey’s post-hoc multiple comparisons test for depicted bar graphs. Experiments are representative of 3 independent experiments.

In order to determine if resveratrol-mediated alterations in inflammation is associated with changes in gut microbiome, we first analyzed the gut microbiota from all experimental groups by using 16S rRNA V3-V4 sequencing technique for microbial profiling. From colonic fecal matter, we isolated genomic DNA and performed pyrosequencing with Illumina MiSeq platform. Nephele analysis output showed that the alpha diversity, represented as chao1, was slightly enriched in the AOM and AOM + Resveratrol groups compared to controls (Figure 2A). In terms of beta diversity depicted as a principle component analysis (PCA) plot, samples clustered within their own respective groups, with resveratrol-treated groups showing more similar diversity compared to naïve controls and the AOM disease group clustering further away from all other experimental groups (Figure 2B). 16s rRNA sequencing analysis from Nephele allowed sample reads to be classified into OTUs from the phylum to the species level (Figure S6), and divergent microbial composition among the experimental groups was apparent starting even at the phylum level. At this taxa level, *Verrucomicrobia* and *Tenericutes* were found to be significantly reduced in abundance within AOM groups compared to the controls, whereas levels of these phyla were restored or increased in AOM + Resveratrol mice (Figure S6). AOM mice also had a significant increase in *Proteobacteria*, which were reduced to similar levels as the control naïve mice in the AOM + Resveratrol treatment group (Figure S6). In a continuing trend, subsets of these altered phylum could be traced down to the genus level (Figure S6). At genus level *Ruminococcus*, *Akkermansia*, *Dehalobacterium*, *Anerostipes*, *Anaeroplasma*, *Blautia*, and *Clostridium* were reduced in AOM mice compared to controls but were restored or increased significantly after treatment with resveratrol (Figure S6), whereas *Oscillospira* and *Desulfovibrio* increased in AOM but were significantly reduced in AOM + Resveratrol groups (Figure S6). As several bacteria were altered in the disease and treated states, LEfSe analysis, which is a useful tool to determine potential bacterial biomarkers among experimental groups [43], was used to highlight the more relevant significant differences in the microbial community. From this analysis, it was found that among the species detected by 16S rRNA sequencing, *Ruminococcus gnavus*, *Akkermansia muciniphillia*, and *Mucispirillum schaedleri* were among the potential biomarkers in the AOM + Resveratrol treatment group (Figure 2C,D). As shown in Figure 2E, all of these species were significantly reduced in AOM mice but were increased after treatment with resveratrol and were validated using PCR (Figure 2F).

In addition to microbial profiling, we investigated the resulting changes in the microbial community altered bacterial-related metabolism, as PiCRUSt allows evaluation of bacterial function to be performed with 16S rRNA data [49]. Using combined PiCRUSt (via Nephele) and LEfSe analyses, it was shown that there were marked changes in microbial functions amongst the experimental groups particularly after treatment with resveratrol, which included those that were connected to CRC (e.g., P53 signaling) [50] and those involved in generation of Tregs (e.g., TGF-β signaling) [51] (Figure S7). Lastly, we determined if changes in SCFA production, metabolite produced by gut flora in the host organism [52], could be seen in response to these changes in the gut microbiome composition triggered by treatment of CRC with resveratrol. Examination of colonic contents showed that n-butyric and i-butyric acid concentrations were significantly reduced in the AOM groups compared to controls. However, AOM group treated with resveratrol restored or increased the levels of these SCFAs (Figure 2G). Among the other detectable SCFAs, propionic acid, i-valeric acid, and n-valeric acid showed no significant changes among the experimental groups (Figure 2G).

Lastly, association studies between changes in the individual species altered by resveratrol treatment during CRC (*Ruminococcus gnavus*, *Akkermansia muciniphillia*, and *Mucispirillum schaedleri*) and other variables, such as disease parameters, immune responses, and n-/i-butyric acid levels, were performed to determine if changes in these species could account for some of these observed results. The abundance of *Ruminococcus gnavus* was shown to have a significant negative correlation with tumor numbers and disease score (Figure S8a,b). The abundance of this species was also found to have a significantly high positive correlation with increasing levels of CD4 + FoxP3 + Tregs and IL-10-producing T cells (Figure S8c,d), whereas it trended with a negative correlation with the inflammatory T cell subsets (Figure S8e,f). There were no significant correlations between *Ruminococcus gnavus* abundance and n-butyric or i-butyric levels however (Figure S8g,h). *Akkermansia muciniphillia* alterations were found to have significant negative correlation with tumor number and disease score (Figure S9a,b), but this species did not have any significant correlations to the anti-inflammatory T cell response (Figure S9c,d). However, there were significant negative correlations with the proinflammatory T cell response and *Akkermansia muciniphillia* levels (Figure S9e,f). Results failed to establish a link between n-butyric or i-butryic levels and the abundance of this species (Figure S9g,h). *Mucispirillum schaedleri* levels also had significant negative correlations with tumor number and disease score (Figure S10a,b), but there was no established association with the levels of this species and the anti-inflammatory T cell responses (Figure S10c,d). There was a significant negative correlation between *Mucispirillum schaedleri* and Th17 numbers (Figure S10e), but no association was established with the Th1 response (Figure S10f). Interestingly, however, there was a significant positive correlation between *Mucispirillum schaedleri* abundance and i-butyric acid concentration (Figure S10g) but not with n-butyric concentrations (Figure S10h). Together, these studies demonstrated that treatment of CRC-induced mice with resveratrol leads to significant changes in both the gut microbial profile and function, which were shown to have significant associations with other variables, such as disease severity and the immune response.

### 3.4. Fecal Transfer from Resveratrol-Treated Groups Attenuates AOM/DSS-Induced CRC and Alters the T Cell-Specific Immune Response

**Figure 3:**
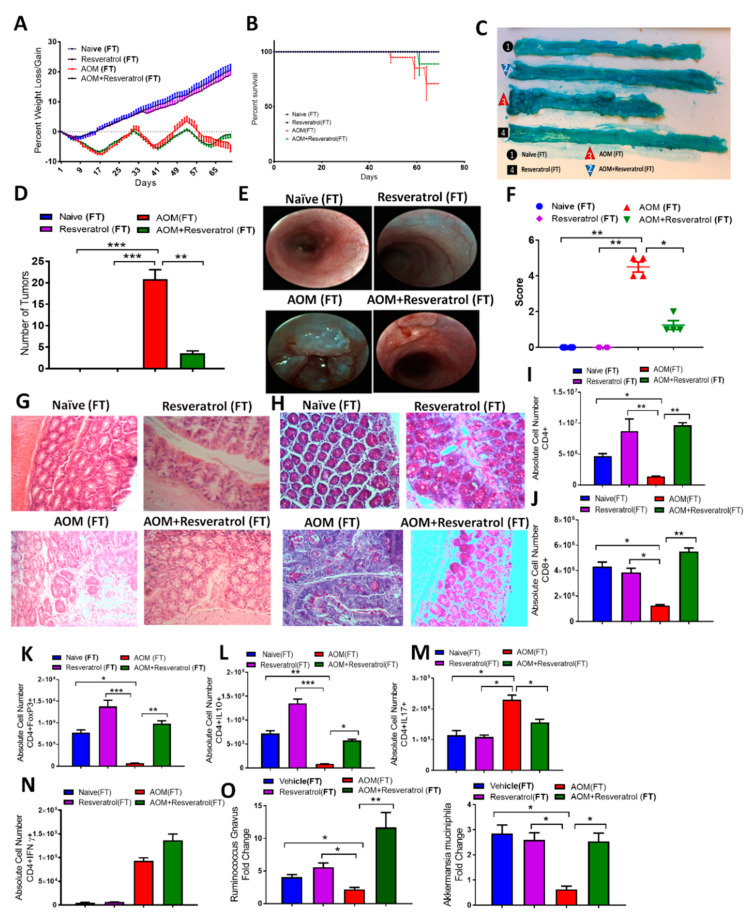
Results from fecal transfer (FT) experiments in AOM-induced CRC model. Antibiotic-treated C57BL/6 mice were injected i.p. with 10 mg of AOM to induce colorectal cancer followed by 3 cycles of 2% DSS. Fecal material was inoculated into recipient mice from the following donors: Naïve (*n* = 4), Resveratrol (*n* = 4), AOM (*n* = 4), and AOM+Resveratrol (*n* = 4). Clinical parameters consisted of percent weight loss (**A**) and survival (**B**), both of which were found to have significant differences in AOM (FT) vs. AOM + Resveratrol (FT) groups. (**C**) Representative colons stained with 1% Alcian blue. (**D**) Bar graph depicting number of tumors counted in each experimental group. (**E**) Representative colonoscope images from experimental groups. (**F**) Bar graph depicting scores after examination of tumor polyps detected during colonoscopies. (**G**) Representative colon sections stained with H&E; scale bar = 100 µM at 40x objective. (**H**) Representative colon sections that underwent PAS staining; scale bar = 100 µM at 40x objective. (**I**,**J**) Bar graphs depicting absolute cell numbers in MLN for general T cells T helper (**I**) and cytotoxic (**J**) T cells. (**K**–**N**) Bar graphs depicting absolute cell numbers in MLN for Tregs (**K**), Th cells producing IL-10 (**L**), Th17 (**M**), and Th1 (**N**) cells. (**O**) PCR validation for the bacterial species *Ruminococcus gnavus* and *Akkermansia muciniphila*. Significance (*p*-value: * *p* < 0.05, ** *p* < 0.01, *** *p* < 0.005) was determined by using one-way ANOVA and post-hoc Tukey’s test for bar/dot graphs, Mann–Whitney test for weight data, and log rank test for survival curve. Data are representative of at least 3 independent experiments.

In order to determine whether or not resveratrol-induced alterations in the gut microbiome were contributing to the altered immune response in CRC, we performed fecal transfer (FT) experiments. After receiving antibiotics to deplete the existing gut microbiome (Figure S11a), AOM-induced CRC recipient mice were inoculated with feces from either Naïve, Resveratrol, AOM, or AOM + Resveratrol groups. Mice inoculated with fecal material from disease controls, referred to as AOM(FT), showed gradual decrease in body weight throughout the study compared to controls, whereas AOM + Resveratrol (FT) mice recovered and gained weight by the end of 10 weeks (Figure 3A). AOM + Resveratrol (FT) mice also showed increased survival when compared to AOM(FT) mice (Figure 3B), along with decreased incidence of tumor development in the colon (Figure 3C,D). In addition, weekly colonoscopy examination (Figure S11b–f) showed increased ulceration and sloughing in portions of the colon in mice fed disease-derived feces; however, mice that were given FT from resveratrol-treated groups showed a reduced presence of polyps and abnormal colonic tissue growths at the end of the study (Figure 3E,F). Histological examination by H&E stains revealed AOM(FT) colon tissues had abnormal growth and damage to the mucosal layer, whereas AOM + Resveratrol tissues resembled that of control colons (Figure 3G). PAS stains also showed that AOM(FT) recipients had decreased mucus production and goblet cells, and just as with treatment with resveratrol in the AOM disease state, AOM + Resveratrol (FT) colon tissues had restored intestinal architecture with normal mucus present and distribution of goblet cells (Figure 3H). Together, these FT experiments demonstrated that the clinical benefits provided by resveratrol against AOM-induced CRC can be attributed, at least in part, to changes in gut microbiota. Next, we tested if the changes in the gut microbiota induced by resveratrol also resulted in changes in inflammation.

As in the previous experiment, flow cytometry analysis was performed in the FT experiments, and collected data showed in the MLNs (Figure S12) of AOM(FT) mice a marked decrease in both T helper (Figure 3I) and cytotoxic T cells (Figure 3J), whereas AOM + Resveratrol (FT) mice had increased numbers of these cells present in the tissue. Going further in phenotyping the CD4 + T helper phenotypes, AOM mice showed significant decreases in anti-inflammatory Tregs (Figure 3K) and CD4 + IL10-producing cells (Figure 3L), which were significantly increased in all resveratrol-treated groups. On the other hand, proinflammatory Th17 (Figure 3M) and IL17-producing CD4 + T cells (Figure 3N) were found to be significantly higher in AOM(FT) recipients compared to the other FT groups, and while AOM + Resveratrol(FT) recipients had decreased Th17 phenotype, this group was not able to decrease Th1 (CD4 + IFNγ+) cells. In order to confirm the transfer of feces resembled our previous sequencing data, PCR validation was performed showing a similar microbial profile for significantly altered species *Ruminococcus gnavus* and *Akkermansia muciniphilia*, which were decreased in AOM (FT) and increased in the AOM + Resveratrol (FT) group (Figure 3O). Collectively, these data demonstrated that the alterations of microbiome by resveratrol were directly modulating the T cell immune response in AOM-induced CRC, particularly in increasing anti-inflammatory subsets (Tregs and CD4 + IL-10-producers), while decreasing proinflammatory types (Th17 and Th1).

### 3.5. Butyrate Supplementation Attenuates AOM/DSS-Induced CRC and Promotes an Anti-Inflammatory T Cell Response Similar to Resveratrol

**Figure 4:**
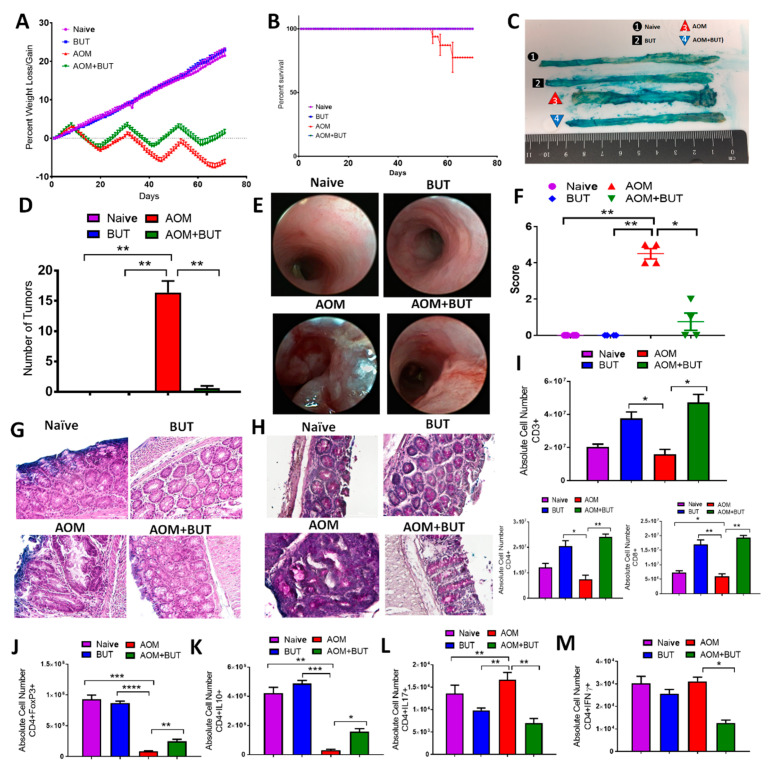
Treatment with sodium butyrate (BUT) reduces clinical symptoms and alters T cell phenotype in AOM-induced CRC model. Female C57BL/6 mice were injected intraperitoneal with 10 mg of AOM to induce colorectal cancer followed by 3 cycles of 2% DSS. Experimental groups consisted of: Naïve (*n* = 4), BUT (*n* = 4), AOM (*n* = 4), and AOM + BUT (*n* = 4). Clinical parameters consisted of percent weight loss (**A**) and survival (**B**), both of which were found to have significant differences in AOM vs. AOM + Resveratrol groups. (**C**) Representative colons stained with 1% Alcian blue. (**D**) Bar graph depicting number of tumors counted in each experimental group. (**E**) Representative colonoscopic images from experimental groups. (**F**) Bar graph depicting scores after examination of tumor polyps detected during colonoscopies. (**G**) Representative colon sections stained with H&E; scale bar = 100 µM at 40x objective. (**H**) Representative colon sections, which underwent PAS staining; scale bar = 100 µM at 40x objective. (**I**) Bar graphs depicting absolute cell numbers in MLN for general T cells (CD3+), T helper (CD3+CD4+), and cytotoxic (CD3+CD8+) T cells. (**J**–**M**) Bar graphs depicting absolute cell numbers in MLN for Tregs (**J**), Th cells producing IL-10 (**K**), Th17 (**L**), and Th1 (**M**) cells. Significance (*p*-value: * *p* < 0.05, ** *p* < 0.01, *** *p* < 0.005, **** *p* < 0.001) was determined by using one-way ANOVA and post-hoc Tukey’s test for bar/dot graphs, Mann–Whitney test for weight data, and log rank test for survival curve. Data are representative of at least 3 independent experiments.

In the current study, one of the distinct outcomes gathered regarding resveratrol-mediated alterations in the gut microbiome was the potential for an increase in SCFA butyrate, which is known to have anti-inflammatory properties [53,54]. Results thus far showed resveratrol could increase colonic butyrate, with modest increases in n-butyric acid and significant increases in i-butyric acid, which has been shown to serve as a precursor for the formation of butyrate [55]. To test its role further, supplementation with sodium butyrate (BUT) was given in lieu of resveratrol to determine the potential effects of increased levels of this SCFA produced in the AOM-induced CRC model. To address this, experimental groups were designed to mimic the previous experiments with the exception of substituting resveratrol with BUT, and these groups included: Naive alone, BUT alone, AOM, and AOM + BUT. As expected, AOM mice had significant decrease in body weight (~20%) compared to controls, but like AOM + Resveratrol groups from the previous experiments, AOM + BUT mice had significant reduction in weight loss over time (Figure 4A). Additionally, while the AOM group had a decrease in overall percent survival, AOM + BUT mice showed 100% survival after 10 weeks (Figure 4B). Similar to AOM + Resveratrol mice, AOM + BUT mice had decreased or nonexistent colonic tumors (Figure 4C,D). Colonoscopic examination at 5 different time points (weeks 0, 3, 5, 7, and 9) during the experiment gave a clear picture of the development of CRC-associated lesions and tissue sloughing after AOM injection, but AOM+BUT groups showed marked decrease in tissue disruption (Figure 4E,F; Figure S13). Histological examination of formalin-fixed colon tissues stained with H&E was also performed on experimental groups, which clearly showed in AOM colons there was a loss of mucosal, submucosal, and serosa architecture, which was not seen in AOM + BUT (Figure 4G). PAS staining showed that AOM + BUT colons were also able to maintain a significant amount of mucus presence and number of goblet cells, which were lost in AOM tissue sections (Figure 4H). Supplementation with butyrate was thus able to attenuate the clinical parameters of AOM-induced CRC much like resveratrol, so it was reasonable to examine whether or not increased butyrate was able to alter the T cell repertoire.

Just as with resveratrol treatment, flow cytometric analysis of the MLN (Figure S14) showed expression of T cells in general, CD4 + T helper, and CD8+ cytotoxic T cells increased in AOM + BUT groups after being depleted in number in AOM-induced CRC (Figure 4I). AOM+BUT groups also had increased numbers in anti-inflammatory Tregs (Figure 4J) and CD4 + IL-10 cells (Figure 4K) when compared to AOM mice that had much lower number of these cells than the control groups. Alternatively, inflammatory Th17 (Figure 4L) and Th1 cells (Figure 4M) were much lower in AOM + BUT mice when compared to the AOM disease controls, in addition to anti-inflammatory CD4 + FOXP3 + and CD4 + IL10 + populations in both mesenteric lymph node and blood. A similar shift from proinflammatory Th17/Th1 to anti-inflammatory Tregs/IL-10 was seen after treatment with BUT in the spleen (Figure S15). Collectively, these data showed that butyrate supplementation reduces the inflammatory T cell response much in the same manner as resveratrol, suggesting that increased production of this SCFA by resveratrol is another mechanism through which this natural compound may be effective against AOM-induced CRC.

### 3.6. Supplementation of Butyrate Alters the Microbial Profile in AOM-Induced CRC With Similarities to Resveratrol Treatment

**Figure 5:**
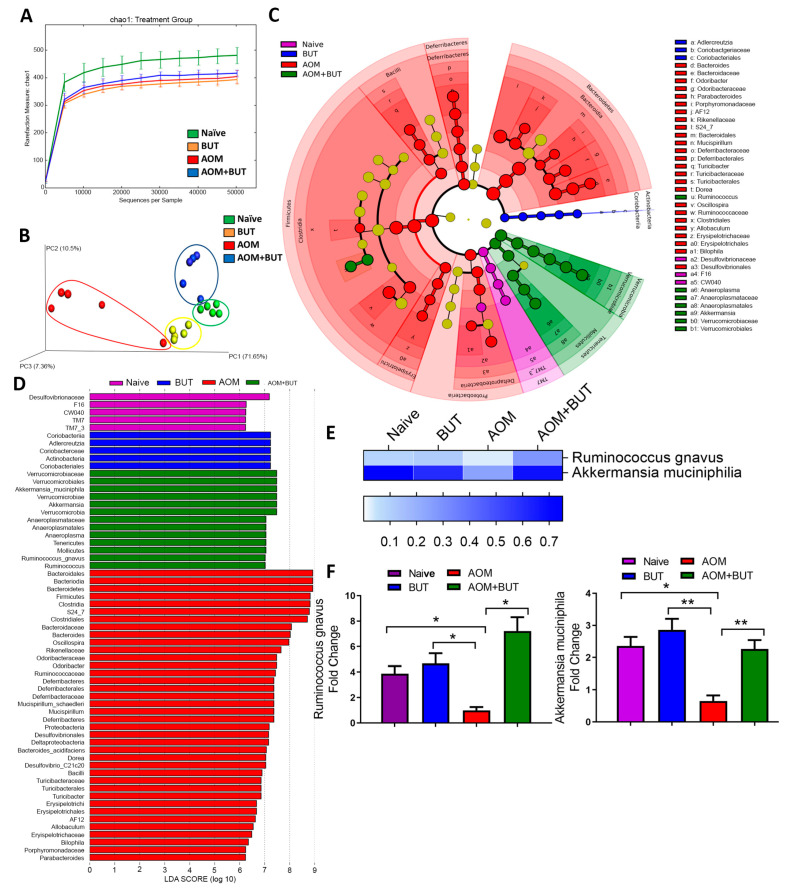
AOM induction and treatment with BUT were performed as described in Figure 4 legend. Gut microbiota samples were collected from experimental groups by performing colonic flushes in experimental groups, which were the following: Naïve (*n* = 5), BUT (*n* = 5), AOM (*n* = 5), and AOM + BUT (*n* = 5). Nephele analysis (nephele.niaid.nih.gov) was used to generate charts for chao1 alpha diversity (**A**) and PCA beta diversity (**B**). LeFSe analysis of the Nephele OTU output files generated the cladogram (**C**) and LDA score bar graph (**D**). (**E**) Heatmap and legend depicting mean OTU percent abundances of significantly altered species *Ruminococcus gnavus* and *Akkermansia muciniphila*. Rows represent bacterial species and columns represent the mean OTU percentages of experimental groups with SEM. (**F**) PCR validation of *Ruminococcus gnavus* and *Akkermansia muciniphila.* Significance (*p*-value: * *p* < 0.05, ** *p* < 0.01) was determined by using one-way ANOVA followed by Tukey’s post-hoc multiple comparisons test for depicted bar graphs. Experiments are representative of 3 independent experiments.

As potential resveratrol-mediated increases in colonic butyrate could also lead to alterations in the microbiome in addition to promoting anti-inflammatory T cell phenotypes, 16S rRNA microbial sequencing was performed on experimental groups in the butyrate supplementation experiments. Alpha diversity indicated that compared to the naïve group, all other experimental groups (BUT, AOM, AOM + BUT) had lower overall diversity within the samples (Figure 5A). Beta diversity or PCA clustering in the butyrate supplementation experiments mimicked closely what was seen in the resveratrol treatment experiments, with all experimental samples clustering within their own groups tightly, but the AOM group being the most divergent (Figure 5B). OTU abundances were calculated from the phylum to the genus level (Figure S16) as previously done. Several significant differences were seen at taxa levels with butyrate supplementation; however, for the current report and in the context of explaining resveratrol-mediated mechanisms, only those with changes similar to the resveratrol treatment experiments are highlighted. For example, BUT-treated groups were able to restore or increase bacteria at the phyla *Verrucomicrobia* and *Tenericutes*, which were significantly reduced in the AOM group, while butyrate supplementation decreased *Proteobacteria* which rose in AOM-induced CRC (Figure S16). As with resveratrol treatment, similar alterations in subsets of these bacterial phyla could be traced down to the genus level (Figure S16). At the genus level and closely mimicking resveratrol treatment experiments, *Ruminococcus*, *Akkermansia*, *Anerostipes*, *Anaeroplasma*, and *Clostridium* were reduced in AOM mice compared to controls but were restored or increased significantly after butyrate supplementation, whereas *Desulfovibrio* increased in AOM but was significantly reduced in AOM + BUT groups (Figure S16). LeFSe analysis was then performed to determine which bacterial species had the highest LDA score among the experimental groups (Figure 5C,D), and combined with OTU abundance data, *Ruminicoccus gnavus* and *Akkermansia muciniphilia* (Figure 5E) were found to be restored by butyrate after depletion in AOM-induced CRC, which was validated with PCR (Figure 5F). Taken altogether, these data suggested that resveratrol-mediated alterations in the gut microbiome and shifting to anti-inflammatory T cell phenotype in AOM-induced CRC can be explained, at least in part, by the ability of resveratrol to increase levels of butyrate in the gut microenvironment.

### 3.7. Resveratrol and BUT Inhibit HDACs In Vivo and In Vitro

**Figure 6:**
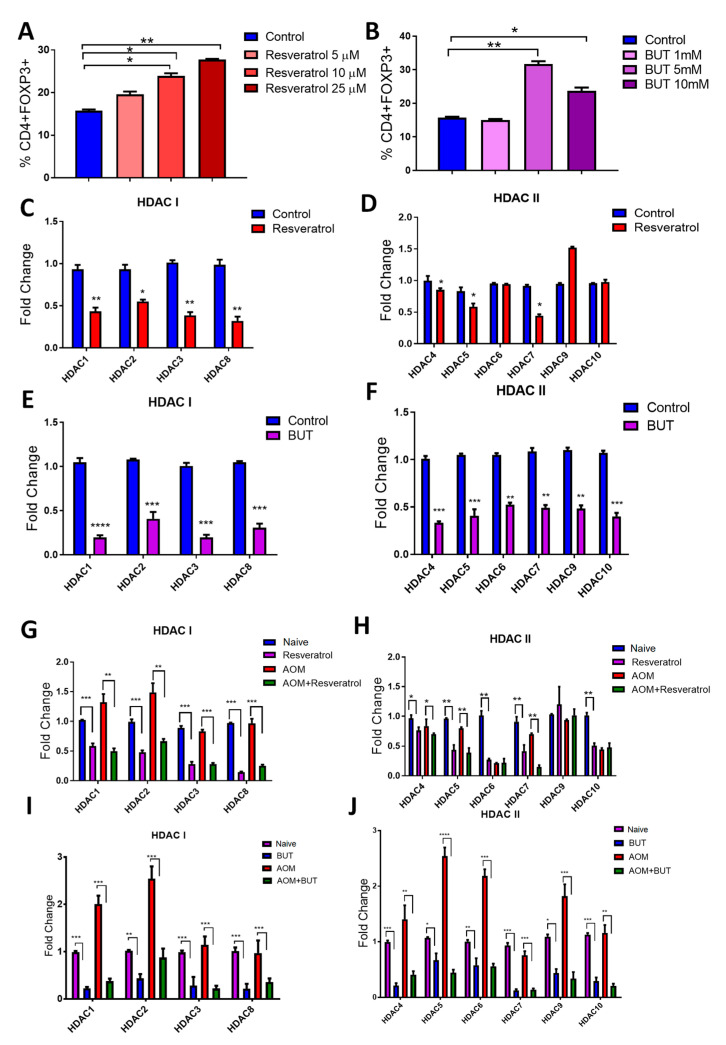
Treatment with Resveratrol and BUT leads to HDAC suppression. Whole splenocytes (seeded at 1 × 106 cells/mL) from 8–10 week old C57BL/6 mice were activated using CD3 (0.5µg/mL) and CD28 (2 µg/mL) in the absence or presence of appropriate vehicle control, resveratrol (5, 10, or 25 µM), or BUT (1, 5, or 10 mM). Tregs were identified by flow cytometry as represented in Figure S17. (**A**) Treg percentages after treatment with various doses of resveratrol. (**B**) Treg percentages after treatment with varying doses of BUT. Fold change expression as assessed by PCR for HDAC I (**C**) and HDAC II (**D**) after treatment with resveratrol (25 µM). Fold change expression as assessed by PCR for HDAC I (**E**) and HDAC II (**F**) after treatment with BUT (5 mM). Expression of HDAC I (**G**) and HDAC II (**H**) was evaluated from MLNs isolated from experimental groups (Naive, Resveratrol, AOM, and AOM+Resveratrol). Expression of HDAC I (**I**) and HDAC II (**J**) was evaluated from MLNs isolated from experimental groups (Naive, BUT, AOM, and AOM+BUT). For in vitro experiments, each group consisted of 3 wells (*n* = 3), and the data are representative of 2 independent experiments. For in vivo experiments, each group consisted of 5 mice (*n* = 5), and the data is representative of at least 3 independent experiments. Significance (*p*-value: * *p* < 0.05, ** *p* < 0.01, *** *p* < 0.005, **** *p* < 0.001) was determined by using one-way ANOVA followed by Tukey’s post-hoc multiple comparisons test for depicted bar graphs.

The increase in colonic butyrate production in resveratrol-treated CRC mice was interesting given the fact that previous reports show butyrate not only increases Treg production [56,57], but the HDAC inhibiting activities of this SCFA have been implicated as a key mechanism in which it exerts anti-inflammatory and anti-cancer properties [58,59,60], including in colorectal cancer models [61]. With this in mind, studies were performed to examine the ability of resveratrol and BUT to suppress HDACs in vitro and in the CRC in vivo model and how this correlated with increased Treg production. For in vitro studies, resveratrol increased Tregs in activated splenocytes in a dose-dependent manner (Figure S17; Figure 6A). BUT significantly increased Tregs at the higher doses (5 and 10mM) when treating activated cells in the same manner (Figure S17; Figure 6B). Following these observations, the expression of class I (HDAC I; HDACs 1, 2, 3, and 8) and class II HDACs (HDAC II; HDACs 4, 5, 6, 7, 9, and 10) were examined in cultures with the most significantly increased Treg expression (25 µM for resveratrol and 5 mM for BUT). Resveratrol in the in vitro cultured system was able to significantly reduce expression of all HDAC I (Figure 6C) but interestingly decreased only selective HDAC II, specifically not being able to reduce HDAC 6, 9, and 10 (Figure 6D). BUT, being a well-known HDAC inhibitor, was able to reduce expression of all HDACs, regardless of specific classes (Figure 6E,F). The expression of HDAC I and II was evaluated in in vivo CRC experiments given treatment with either resveratrol or BUT. Interestingly, similar results were seen. For HDAC I, resveratrol treatment compared to either naïve controls or CRC disease mice resulted in decreased expression (Figure 6G). While HDAC II were all decreased upon treatment with resveratrol compared to naïve controls, once again, select HDACs (HDAC 6, 9, and 10) were not inhibited by resveratrol compared to AOM disease controls (Figure 6H). However, supplementation experiments with BUT resulted in overall decreased HDAC expression for HDAC I and HDAC II (Figure 6I,J). Lastly, association analyses were performed to see if any of the altered species by resveratrol treatment in CRC (*Ruminococcus gnavus*, *Akkermansia muciniphillia*, and *Mucispirillum schaedleri*) could be linked to changes in expression of HDACs (Figures S18–S20). For HDAC I, *Ruminococcus gnavus* was shown to have a significant negative correlation with HDAC 1 and 3 expression levels (Figure S18a), and only a negative correlation with HDAC7 in HDAC II levels (Figure S18b). *Akkermansia muciniphillia* abundances had even fewer associations, as only HDAC 1 (Figure S19a) and HDAC 10 (Figure S19b) levels showed significant negative correlations. Interestingly, *Mucispirillum schaedleri* abundances showed significant negative correlations with all HDAC I levels (Figure S20a), though no significant associations were established with HDAC II levels (Figure S20b). Altogether, this data suggests that while increased butyrate can lead to inhibition of HDACs, which correlates to increased Treg expansion, resveratrol is able to at least in part reduce select HDAC expression itself, independent of butyrate or any alterations of the microbiome after treatment.

### 3.8. Increased Expression of Anti-Inflammatory T Cell Markers Results in Increased Survival in Human CRC Patients

**Figure 7:**
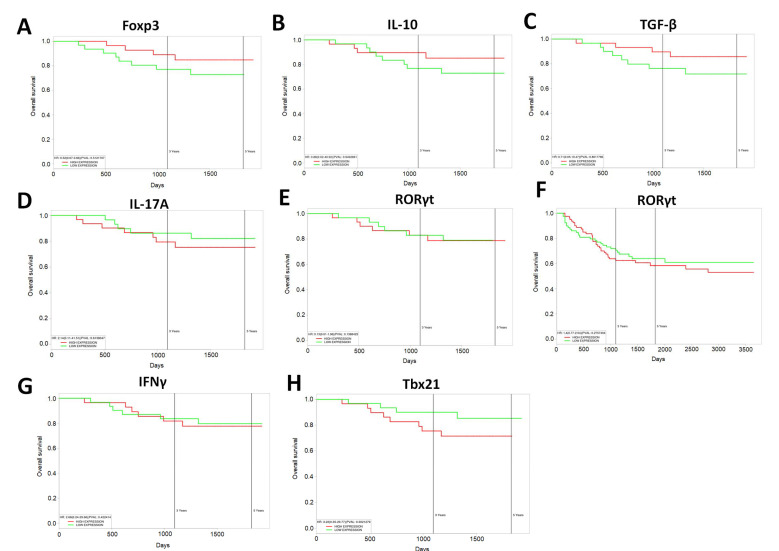
TCGA datasets for colorectal cancer from The Cancer genome Atlas (TCGA, https://cancergenome.nih.gov/) were used to correlate gene expression with patient survival over a 5-year period or more. Correlations to patient survival were performed based on the following gene expressions: (**A**) FoxP3, (**B**) IL-10, (**C**) TGF-β, (**D**) IL-17A, (**E**) ROR-γt, (**F**) ROR-γt (past five year interval), (**G**) IFN-γ, and (**H**) Tbx21. Kaplan–Meier survival curves, defined as the probability of survival in a given length of time while considering time in many small intervals, were used to generate survival curves plots.

Lastly, as the current study was able to show that resveratrol modulated the gut microbiome to increase anti-inflammatory T cell subsets (Treg and IL-10-producers) while decreasing proinflammatory Th17/Th1 types, we examined whether there was any correlation between gene expression of T cell-specific makers in CRC patients with survival. Looking at the TCGA datasets of CRC patients, it was shown that increased expression of Treg-specific transcription factor FoxP3 (Figure 7A) or anti-inflammatory IL-10 (Figure 7B) resulted in an increase in CRC 5-year patient survival. High expression of TGF-β, known to influence the development of Tregs, also correlated with increased overall survival in the patient population (Figure 7C). However, high expression of Th-17 associated IL-17 cytokine was just the opposite, as it resulted in decreased patient survival, while patients with lower expression of Il-17 had increased survival over time (Figure 7D). While expression of Th-17 transcription factor RORγt was shown to have no difference in CRC patient survival over a 5-year period (Figure 7E), expansion past 5 years showed that high expression of this transcription factor was capable of bringing down overall CRC patient survival (Figure 7F). Just as in the AOM-induced mouse model in the current study, IFNγ expression associated with Th1 cells did not seem to have much effect on CRC patient survival (Figure 7G), but in the context of Th1-specific transcription factor (Tbx21), high expression of this gene did appear to decrease the expected overall CRC patient survival. The results in the current study are promising given that T cell differentiation altered by resveratrol towards anti-inflammatory phenotype, via modulation of the gut microbiome, appears to have significant impact in overall human CRC patient survival.

## 4. Discussion

Published reports with resveratrol date back to the late 1970s, and since then research has shown that this natural plant polyphenol has therapeutic properties ranging from anti-inflammatory [62], anti-oxidant [63,64], anti-depressant [65,66], anti-atherogenic [67], anti-aging [68], as well as anti-cancer [69,70,71]. Our lab has published extensively on the anti-inflammatory properties of resveratrol in various disease models, showing often how the effects of this compound are AhR-dependent [37,38,72,73,74]. Activation of AhR by known ligands, such as resveratrol, has been shown by us as well as others to have significant impact on T cell development and phenotype [11,75]. For example, we have shown that activation of AhR by dietary indoles in a delayed-type hypersensitivity (DTH) model is essential for shifting the T cell response from a proinflammatory Th17 to an anti-inflammatory Treg phenotype [76]. This is important in regards to CRC as studies have shown that high expression of Tregs in CRC patients indicate a more favorable prognosis [77,78], whereas increased Th17 has been linked to CRC pathogenicity and tumor development [79,80,81]. The current report reinforces this notion, as gene expression data of CRC patients seemed to indicate that high expression of anti-inflammatory T cell factors (FoxP3, IL-10) improved patient survival, whereas proinflammatory makers linked to Th17 and Th1 phenotypes decreased overall survival in the patient population. It is important to note that in the context of cancer, the exact role of Tregs/Th17 is not so clear, as other reports indicate that Tregs promote cancer development, whereas inflammatory Th17 cells prevent tumor invasion and metastasis [82,83,84]. Reports seem to indicate that the role these T cell phenotypes in cancer largely depends on the type of cancer along with the stage of disease severity, as well as whether the cancer is driven by chronic inflammation, which could explain the conflicted reports. For CRC, which is linked to chronic inflammation in the colon, an anti-inflammatory response may be more favorable, at least in terms of the early stages of the disease. This could explain why our lab as well as others have shown resveratrol as an effective preventative treatment in CRC animal models [19,20,85,86], which is in part due to the ability of this compound to shift from a proinflammatory T cell response to anti-inflammatory one. While the ability of resveratrol to illicit this type of immune response has been well-characterized, the significance of this report is in the fact that resveratrol-mediated modulation of the gut microbiome seems to be an important mechanism in promoting this T cell shift.

A recent report by Wong et al. showed that inoculation with feces from CRC patients in germ-free or conventional mice resulted in an increase in colonic tumor development, proinflammatory markers, and Th17 phenotype [87]. These findings are interesting because in addition to promoting the idea of Th17 as CRC-inducing in nature as discussed already, this report showed that microbiota plays an important role in CRC development and progression. Prior to and since this report, it has been well-established that the complex interaction between microbiota and the host immunity plays major roles in CRC pathogenicity [21,88]. For example, several bacteria, such as *Helicobacter pylori*, *Streptococcus bovis*, *Bacteroides fragilis*, and *Clostridium septicum* have been known to be major contributors to CRC development [89]. Even shifts in certain phylum, such as increases in *Proteobacteria*, have been associated with CRC malignancy [90]. Thus, it would stand to reason that therapeutics directed at combating CRC disease would also be able to modulate the gut microbiome to promote more beneficial effects.

Previous reports have shown that resveratrol was capable of altering the gut microbiome in other disease models [30,91]. In line with the current study, researchers found that resveratrol was able to increase bacteria such as *Verrucomicrobia* and *Akkermansia muciniphila*, while decreasing *Bacteroides*, *Dysgonomonas*, and *Turicibacter* in a hypertension model with high-fructose diet [92]. Additionally, in an obesity model, it was found that resveratrol treatment increased *Akkermansia* and *Ruminococcaceae*, which were shown to alleviate the clinical effects associated with a high-fat diet [28]. Interestingly enough, studies found that *Ruminococcus gnavus* was in fact reduced in CRC patients when compared to controls [93], and that *Akkermansia muciniphilia* was associated with increased response to chemotherapy in CRC patients [94]. The current study showed that these species were decreased in AOM-induced CRC, which correlates well with human CRC patients, but more importantly, resveratrol was able to restore or increase these bacteria. This could further explain why resveratrol is such an effective therapeutic in CRC models, as it seems to increase the presence of bacteria lost or decreased during CRC development and progression. These particular bacteria appear to possess properties essential for controlling and preventing tumor development in the gastrointestinal system.

Alterations in the microbial profile were not the only interesting aspect obtained from the current studies, but rather it was significant to find that resveratrol-mediated alterations in the gut microbiome could promote butyrate production as well. While the current report showed modest increases in n-butyric acid levels, the branched SCFA i-butryic acid (also known as isobutyric acid or 2-methylpropanoic acid) was found to be significantly increased. These results were similar to our previously published report showing resveratrol altered the same SCFAs in a mouse model of colitis [95]. This is significant because isobutyrate, the salt form of i-butyric acid, has been shown to be reduced in ulcerative colitis and Crohn’s Disease patients [96] and act as a precursor to butyrate or n-butyrate [55]. In fact, anaerobic bacteria have been known to convert isobutyrate into butyrate and vice versa [97]. Butyrate has been shown to act in an anti-inflammatory manner in various disease models. For example, supplementation with sodium butyrate was capable of attenuating diabetes-associated inflammation [98] as well as inflammation linked to high-fat-diet-induced non-alcoholic fatty liver disease [99]. Oral administration of butyrate was also shown to reduce microbial-associated gastrointestinal inflammation and liver disease in Gulf War illness by mechanisms such as decreasing inflammatory-mediated toll-like receptor (TLR) activation [100]. In the context of CRC specifically, decreases in butyrate production are linked to CRC development [101], and it was shown that butyrate inhibited aberrant epigenetic modifications in CRC cells by upregulating α-ketoglutarate, which is important in mediating DNA methylation [102]. A more recent report closely linked to the current one showed that a mix of SCFA (butyrate, acetate, and propionate) was protective against AOM-induced CRC and was able to suppress key inflammatory cytokines such as IL-6 and inducing apoptosis in tumor-associated epithelial cells [103]. However, in our studies, we focused solely on butyrate supplementation and showed that this SCFA could alter T cells in CRC specifically by shifting from inflammatory Th1/Th2 to anti-inflammatory Treg/IL-10-producers. One of the key mechanisms through which butyrate has been shown to promote an anti-inflammatory response is inhibition of HDACs [104,105], even in the case of promoting Treg production specifically in CRC [61]. Interestingly though, the results in the current report seemed to suggest that resveratrol alone was promoting HDAC inhibition, independent of butyrate, and this correlated with induction of Tregs. However, it is important to note that resveratrol treatment in CRC provided potential evidence of increased colonic butyrate levels, and the T cell subsets were examined in the gut-specific draining lymph node (MLN). Therefore, the relationship between resveratrol modulation of the gut microbiome, possible increases in butyrate, HDAC expression, and Treg expansion needs to be further analyzed at the local site, in this case the colon, to further understand the interplay related to these mechanisms.

Combined with the findings discussed above, the current study is able to provide new and exciting insights into how resveratrol has the potential to be a strong preventive agent against CRC. As the gut microbiota is now known to be important in disease progression and development, the fact that resveratrol can modulate this microenvironment in such a way as to induce a beneficial T cell immune response proven to help CRC patients is important, and through FT experiments, this resveratrol-mediated mechanism linked to microbiome-modulation appears more conclusive now. In addition, to our knowledge, this is the first report to confirm this and also provide evidence that resveratrol modulates the microbiome to increase butyrate production. Our studies also suggest that resveratrol and other dietary AhR ligands may constitute preventive modalities in the fight against CRC and potentially other types of inflammatory diseases linked to microbial dysbiosis.

## Figures and Tables

**Table 1:** Primer sequences.

Primer	Forward	Reverse
*Ruminococcus gnavus*	AGAGGGATGTCAAGACCAGGTA	TACTAGGTGTCGGGTGGAAAAG
*Akkermansia muciniphila*	GTATCTAATCCCTTTCGCTCCC	GACTAGAGTAATGGAGGGGGAA
*Mucispirillum schaedleri*	CACATGCAAGTCAGGGAGAAA	CAGGTCTCCCCAACTTTTCCTA
HDAC 1	CCGCATGACTCACAATTTGCT	TCTGGGCGAATAGAACGCAGG
HDAC 2	TACAACAGATCGCGTGATGAC	TCCCTTTCCAGCACCAATATC
HDAC 3	GAAATGTTGCCCGGTGTTGGA	TGAGTTCTGATTCTCGATGCG
HDAC 4	AACTTCTTCCCAGGAAGTGGA	TGCGATAGGCATAACCACCGT
HDAC 5	TGGACTGGGATATTCACCATG	AGAGCCTGGAAAGAAGTTCCC
HDAC 6	ATTGCTGCTTTCCTGCACATC	AATCAACTTGCCTCCTGCCAA
HDAC 7	GCTGAAGAATGGCTTTGCTGT	AATGAGGATCTTGCTGGCTTT
HDAC 8	AGTGCCTGATTGACGGGAAGT	CGGTCAAATTTCCGTCGCAAT
HDAC 9	AGGATGATGATGCCTGTGGTG	GCCTGGTCAAATTCTGGTGCT
HDAC 10	AGCAGAAATATGGGCTGAAGA	AGAAGCTTCCATGCTCATAGC

## Supplementary Materials

The following are available online at https://www.mdpi.com/2077-0383/9/6/1796/s1. Figure S1: Weekly colonoscopy images in AOM-induced CRC treated with resveratrol. Figure S2: T cell phenotyping in MLN of AOM-induced CRC mice treated with resveratrol. Figure S3: T cell phenotyping in spleen of AOM-induced CRC mice treated with resveratrol. Figure S4: T cell phenotyping in blood of AOM-induced CRC mice treated with resveratrol. Figure S5: MDSCs in the spleen and blood of AOM-induced CRC mice treated with resveratrol. Figure S6: Significantly altered bacteria in AOM-induced CRC sample treated with resveratrol at the phylum to genus levels. Figure S7: LefSe analysis of Nephele-generated PiCRUSt data investigating bacterial function based on 16S rRNA sequencing. Figure S8: Correlation between *Ruminococcus Gnavus* abundance and clinical parameters. Figure S9: Correlation between *Akkermansia muciniphila* abundance and clinical parameters. Figure S10: Correlation between *Mucispirillum schaedleri* abundance and clinical parameters. Figure S11: Validation and weekly colonoscopy images in FT experiments. Figure S12: T cell phenotyping in MLN of FT experiments. Figure S13: Weekly colonoscopy images in AOM-induced CRC treated with BUT. Figure S14: T cell phenotyping in MLN of AOM-induced CRC mice treated with BUT. Figure S15: T cell phenotyping in spleen of AOM-induced CRC mice treated with BUT. Figure S16: Significantly altered bacteria in AOM-induced CRC sample treated with BUT at the phylum to genus levels. Figure S17: Resveratrol and BUT dose-dependently increase Tregs in vitro. Figure S18: Correlation between *Ruminococcus gnavus* abundance and HDAC expression. Figure S19: Correlation between *Akkermansia muciniphilia* abundance and HDAC expression. Figure S20: Correlation between *Mucispirillum schaedleri* abundance and HDAC expression.
