# Supplementary material for: Alterations in the Gut Microbiome and Suppression of Histone Deacetylases by Resveratrol Are Associated with Attenuation of Colonic Inflammation and Protection Against Colorectal Cancer
Source: J Clin Med. 2020 Jun 9;9(6):1796. doi: 10.3390/jcm9061796 (PMC7355848; doi:10.3390/jcm9061796)
Supplement: Supplementary file 1 [file jcm-09-01796-s001.pdf]

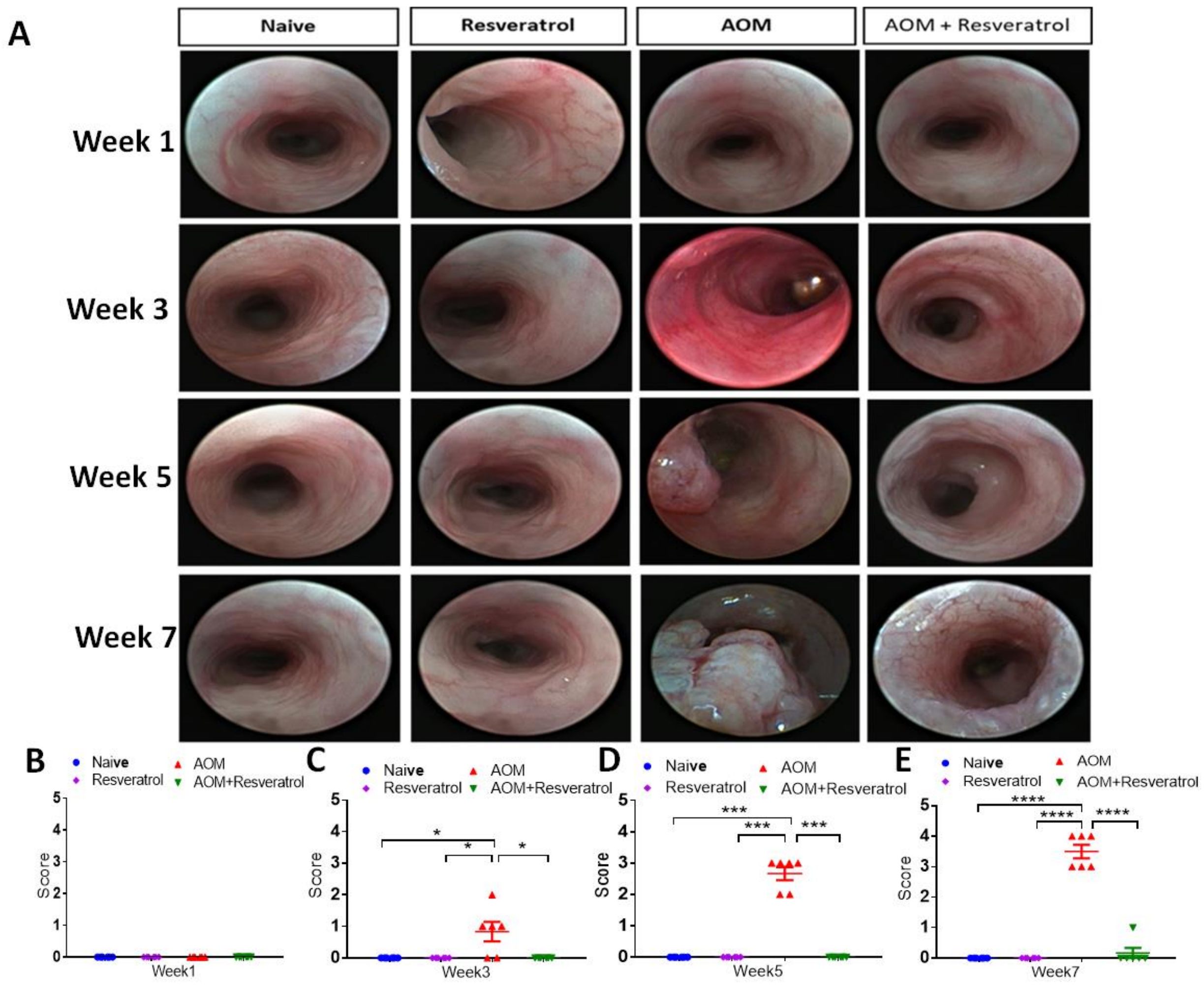

Figure S1. Weekly colonoscopy images in AOM-induced CRC treated with resveratrol. Induction of AOM CRC and treatment with resveratrol were performed as described in Figure 1 legend. (**a**) Representative colonoscopies are shown from experimental groups which included Naïve (n=6), Resveratrol (n=6), AOM (n=6), AOM+Resveratrol (n=6) at weeks 1, 3, 5, and 7. Bar graphs depict colonoscopy scores (described in Materials and Methods) for experimental groups at week 1(**a**), week 3 (**c**), week 5 (**d**), and week 7 (**e**). Significance (p-value: *<0.05, **<0.01, ***<0.005, ****<0.001) was determined by using one-way ANOVA followed by Tukey's post-hoc multiple comparisons test for depicted bar graphs.

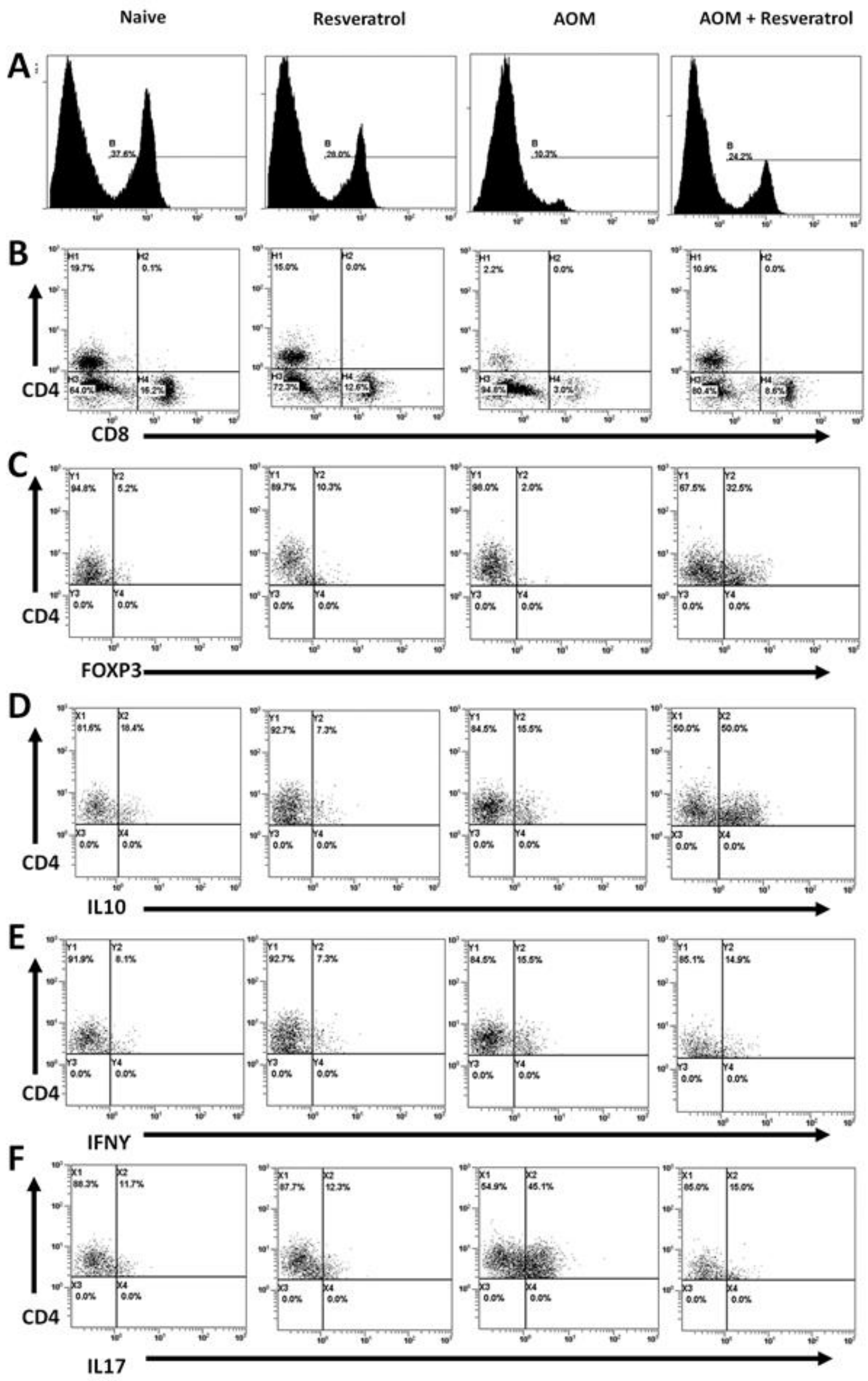

Figure S2. T cell phenotyping in MLN of AOM-induced CRC mice treated with resveratrol. Induction of AOM CRC and treatment with resveratrol was performed as described in Figure 1 legend. Representative flow cytometry histograms and dot plots are depicted for the following T cell subsets: CD3+ (**a**), CD3+CD4+CD8+ (**b**), CD4+FOXP3+ (**c**), CD4+IL10+ (**d**) and CD4+IFNγ+ (**e**), and CD4+IL-17+ (**f**). For Figures c-f, cells were gated on CD4+ population. Data are representative of at least 3 independent experiments.

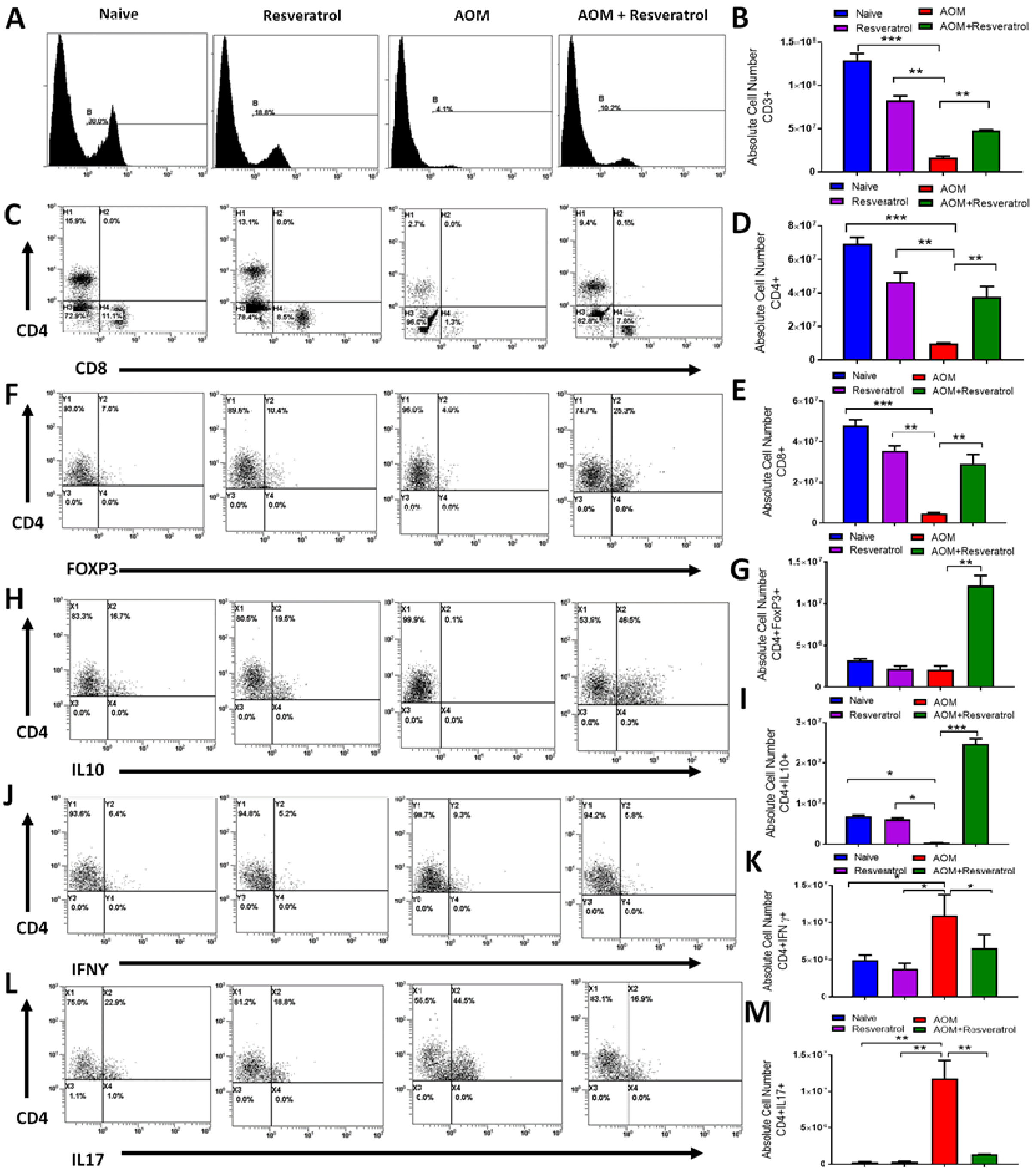

Figure S3. T cell phenotyping in spleen of AOM-induced CRC mice treated with resveratrol. Induction of AOM CRC and treatment with resveratrol was performed as described in Figure 1 legend. Flow cytometry dot plots and quantitative bar graphs depicting absolute cell numbers are shown respectively for the following T cell subsets: CD3+ (**a-b**), CD4+ or CD8+ cells (**c-e**), CD4+FOXP3+ (**f-g**), CD4+IL10+ (**h-i**) and CD4+IFNγ+ (**j-k**), and CD4+IL-17+ (**l-m**) expressing cells. For Figures F-M, cells were gated on CD4+ population. Each experimental group had at least 5 mice included, and significance (p-value: *<0.05, **<0.01, ***<0.005, ****<0.001) was determined for absolute cell numbers by using one-way ANOVA followed by Tukey's post-hoc multiple comparisons test. Data are representative of at least 3 independent experiments.

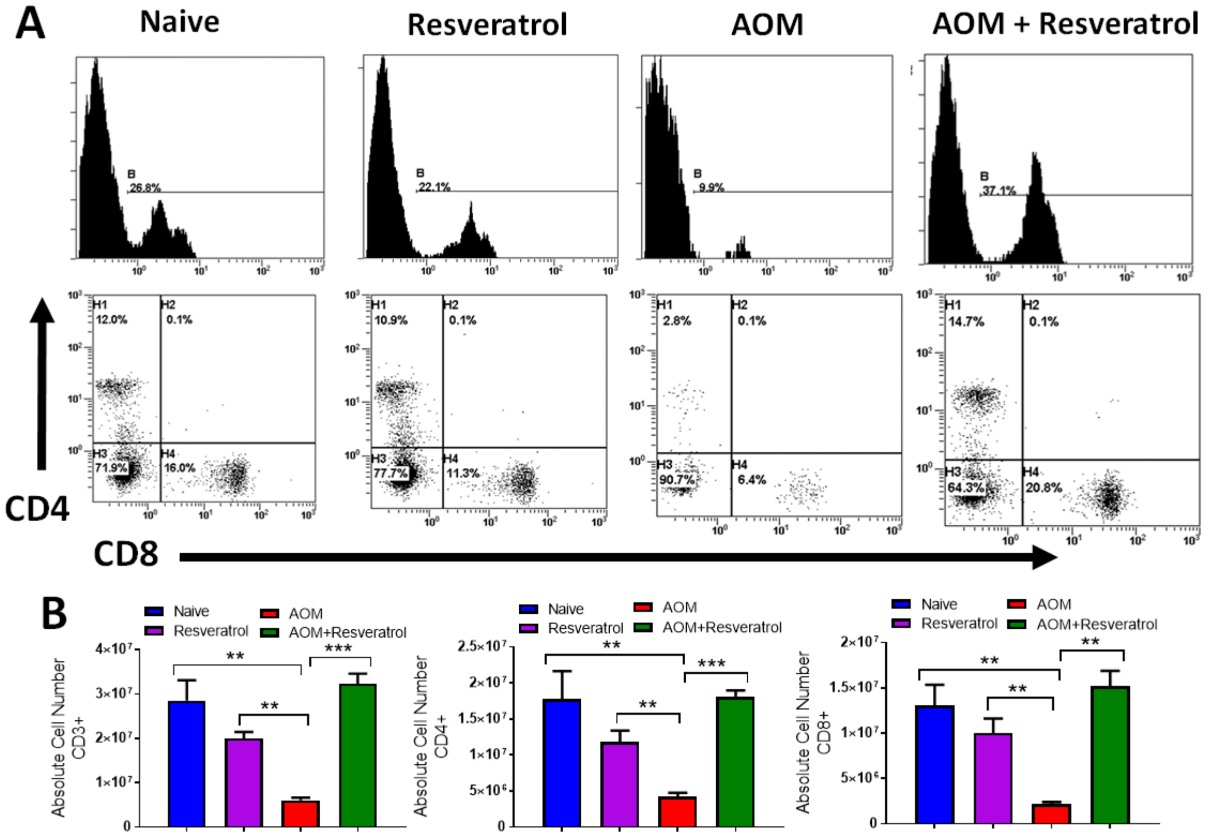

Figure S4: T cell phenotyping in blood of AOM-induced CRC mice treated with resveratrol. Induction of AOM CRC and treatment with resveratrol was performed as described in Figure 1 legend. (**a**) Representative flow cytometry plots are depicted showing CD3+ histogram (top) and CD4+/CD8+ dot plots (bottom) from experimental groups. (**b**) Bar graphs showing absolute cell numbers for the following T cell subsets respectively: CD3+, CD3+CD4+ (T helper), and CD3+CD8+ (cytotoxic T cells). Significance (p-value: *<0.05, **<0.01, ***<0.005, ****<0.001) was determined by using one-way ANOVA followed by Tukey's post-hoc multiple comparisons test in bar graphs. Experiments are representative of at least 3 independent experiments.

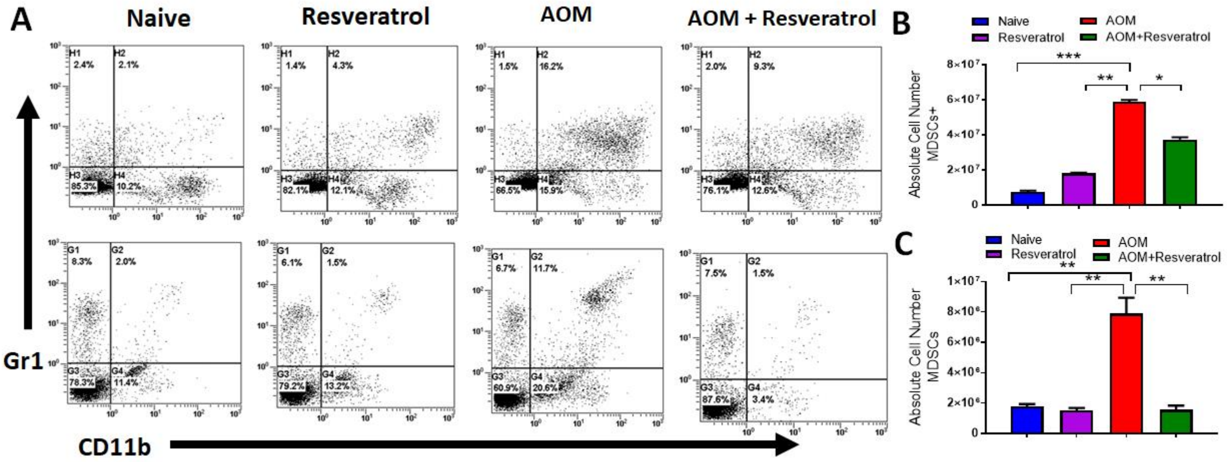

Figure S5. MDSCs in the spleen and blood of AOM-induced CRC mice treated with resveratrol. Induction of AOM CRC and treatment with resveratrol was performed as described in Fig. 1 legend. (**a**) Representative flow cytometry dot plots are depicted showing MDSCs (CD11b+GR1+) in the spleen (top) and blood (bottom) from experimental groups. (**b**) Bar graphs showing absolute cell numbers for MDSCs in the spleen. (**c**) Bar graphs showing absolute cell numbers for MDSCs in the blood. Significance (p-value: *<0.05, **<0.01, ***<0.005, ****<0.001) was determined by using one-way ANOVA followed by Tukey's post-hoc multiple comparisons test in bar graphs. Experiments are representative of at least 3 independent experiments.

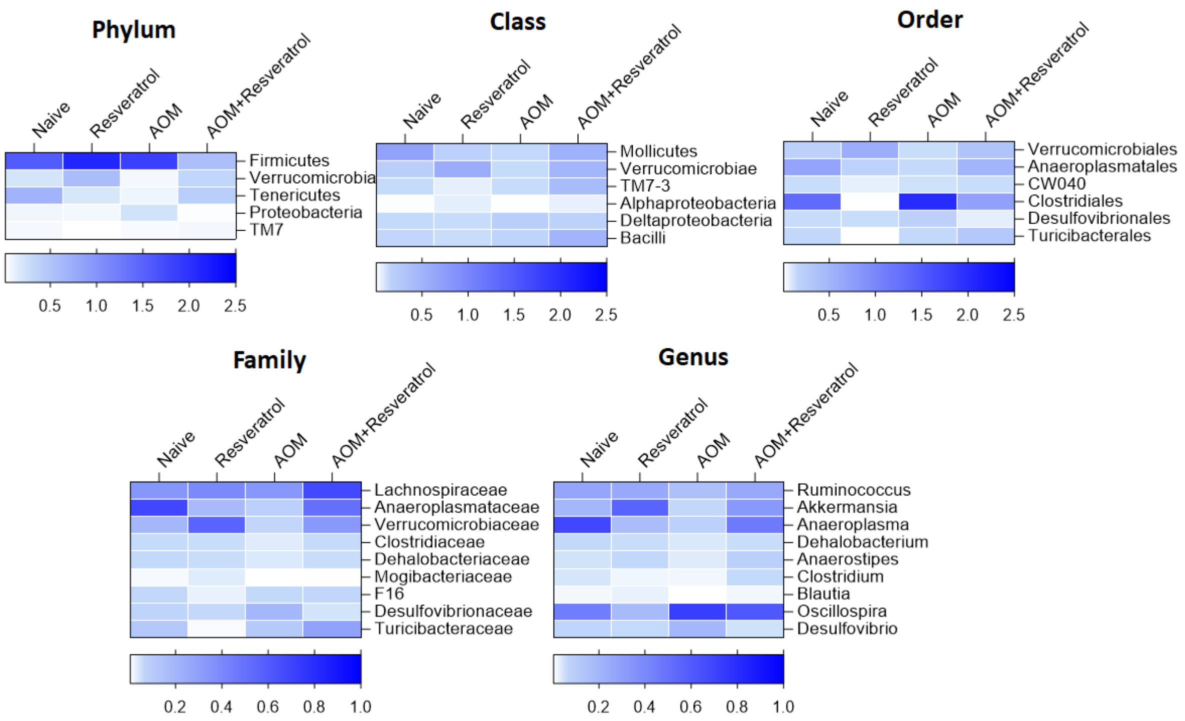

Figure S6. Significantly altered bacteria in AOM-induced CRC sample treated with resveratrol at the phylum to genus levels. Induction of AOM CRC and treatment with resveratrol was performed as described in Figure 1 legend, and 16S rRNA sequencing was performed as described in Figure 2 legend. Bacteria depicted in rows were found to be significantly different (p-value: <0.05) between one or more of the experimental groups using one-way ANOVA and Tukey's multiple comparisons test. Columns represent the experimental groups: : Naïve (n=7), Resveratrol (n=9), AOM (n=10), and AOM+Resveratrol (n=9), and each cell represents the mean OTU abundance of the corresponding bacteria with SEM.

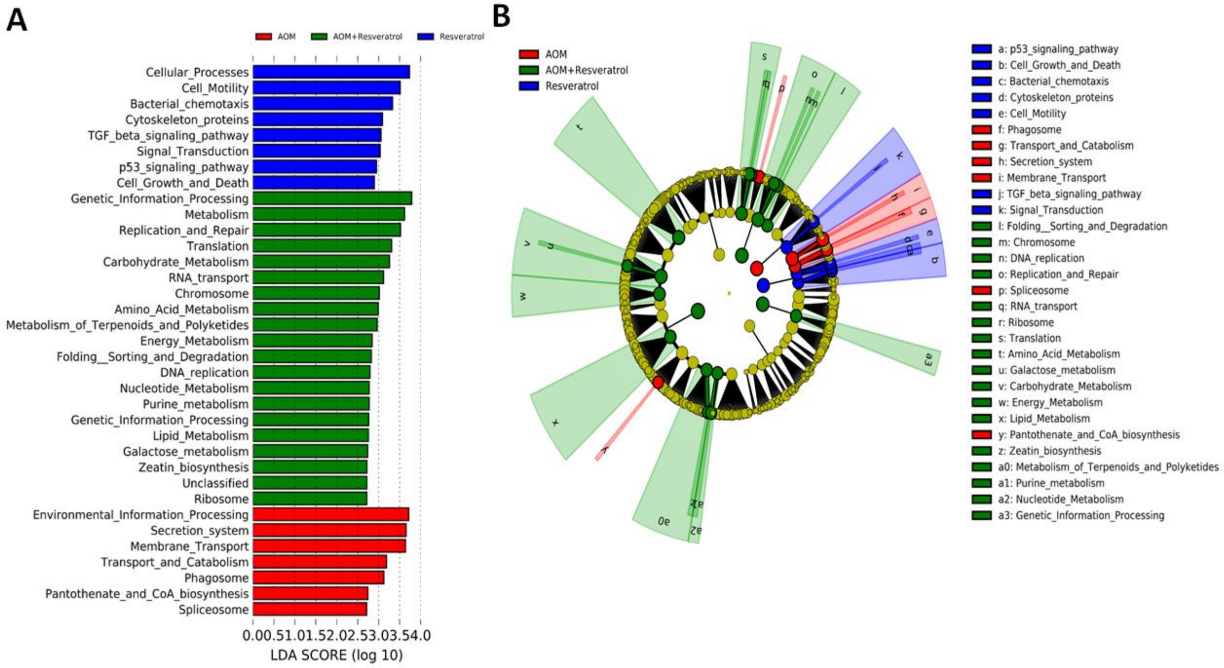

Figure S7. LefSe analysis of Nephele-generated PiCRUSt data investigating bacterial function based on 16S rRNA sequencing. To study functional changes within the microbial samples collected, Nephele-based was performed using the PiCRUSt option, which requires a closed reference against the Greengenes database (Greengene_99) at taxa levels 2 and 3 for KEGG annotations of the uploaded dataset. LefSe was performed using the OTU table generated from the Nephele output analysis software as described in Materials and Methods. Results depicting (**a**) LDA Score, and (**b**) Cladogram are shown.

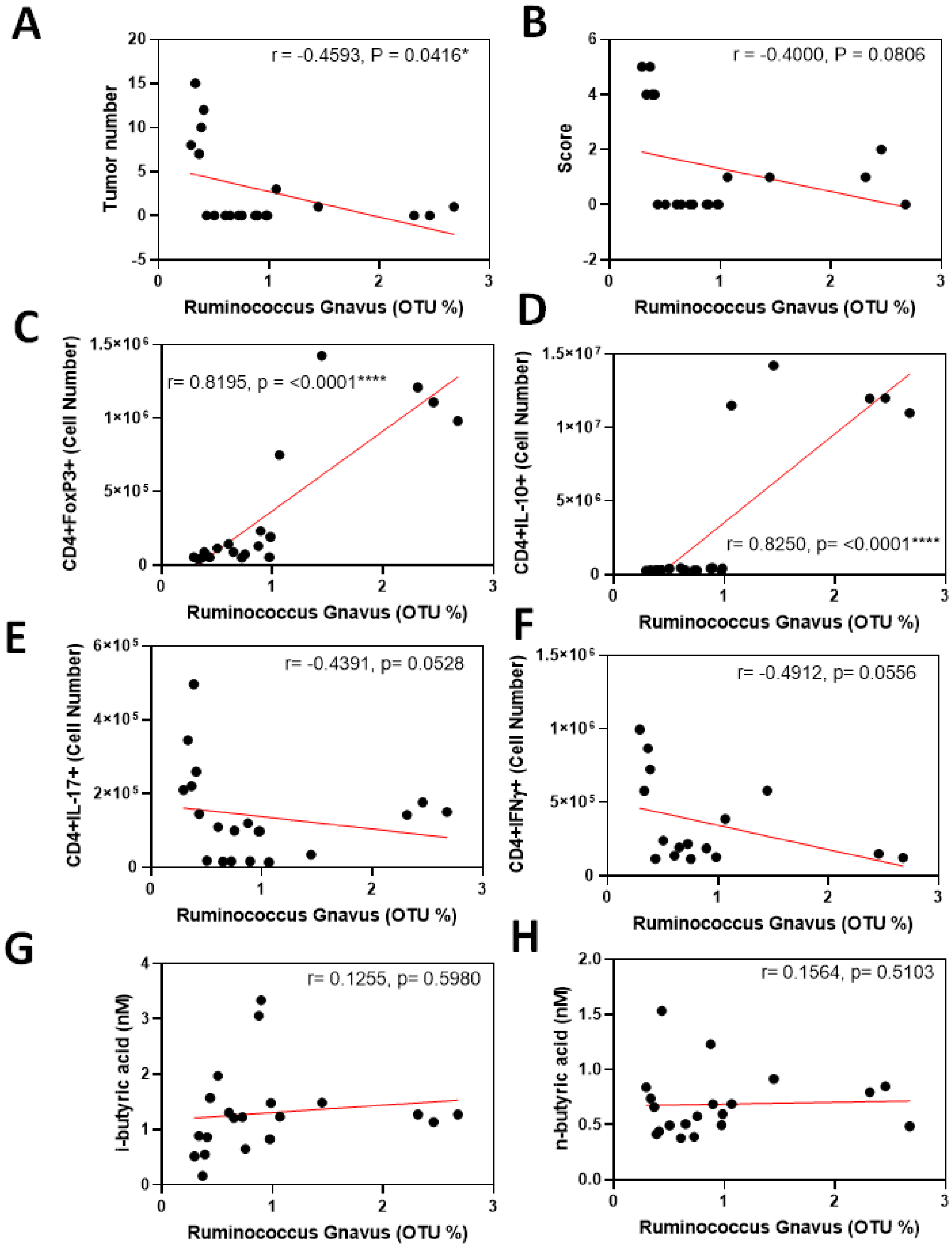

Figure S8: Correlation between Ruminococcus gnavus abundance and clinical parameters. Spearman correlations were performed between OTU percentages of Ruminococcus gnavus from experimental groups (Naïve, Resveratrol, AOM, AOM+Reservatrol; n=5 per group) to other variables to include (**a**) tumor number, (**b**) disease score, (**c-f**) Th subsets, and (**g-h**) i-/n-butyric concentrations. Linear regression line (red), rank coefficient (r), and p-values (p) are depicted in the boxes of each comparison. Significance was determined as follows: p-value: *<0.05, **<0.01, ***<0.005, ****<0.001.

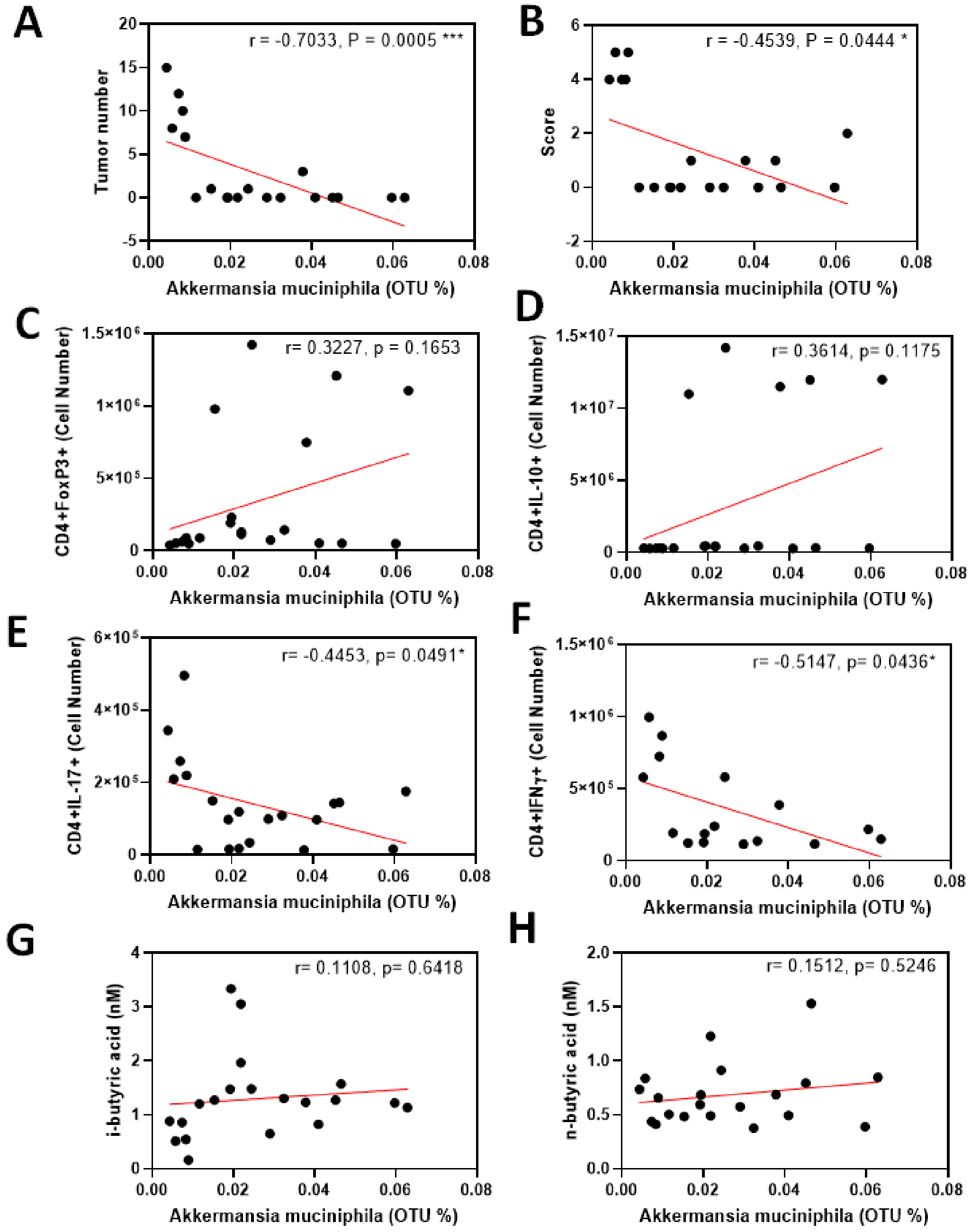

Figure S9. Correlation between Akkermansia muciniphila abundance and clinical parameters. Spearman correlations were performed between OTU percentages of Akkermansia muciniphila from experimental groups (Naïve, Resveratrol, AOM, AOM+Reservatrol; n=5 per group) to other variables to include (**a**) tumor number, (**b**) disease score, (**c-f**) Th subsets, and (**g-h**) i-/n-butyric concentrations. Linear regression line (red), rank coefficient (r), and p-values (p) are depicted in the boxes of each comparison. Significance was determined as follows: p-value: *<0.05, **<0.01, ***<0.005, ****<0.001.

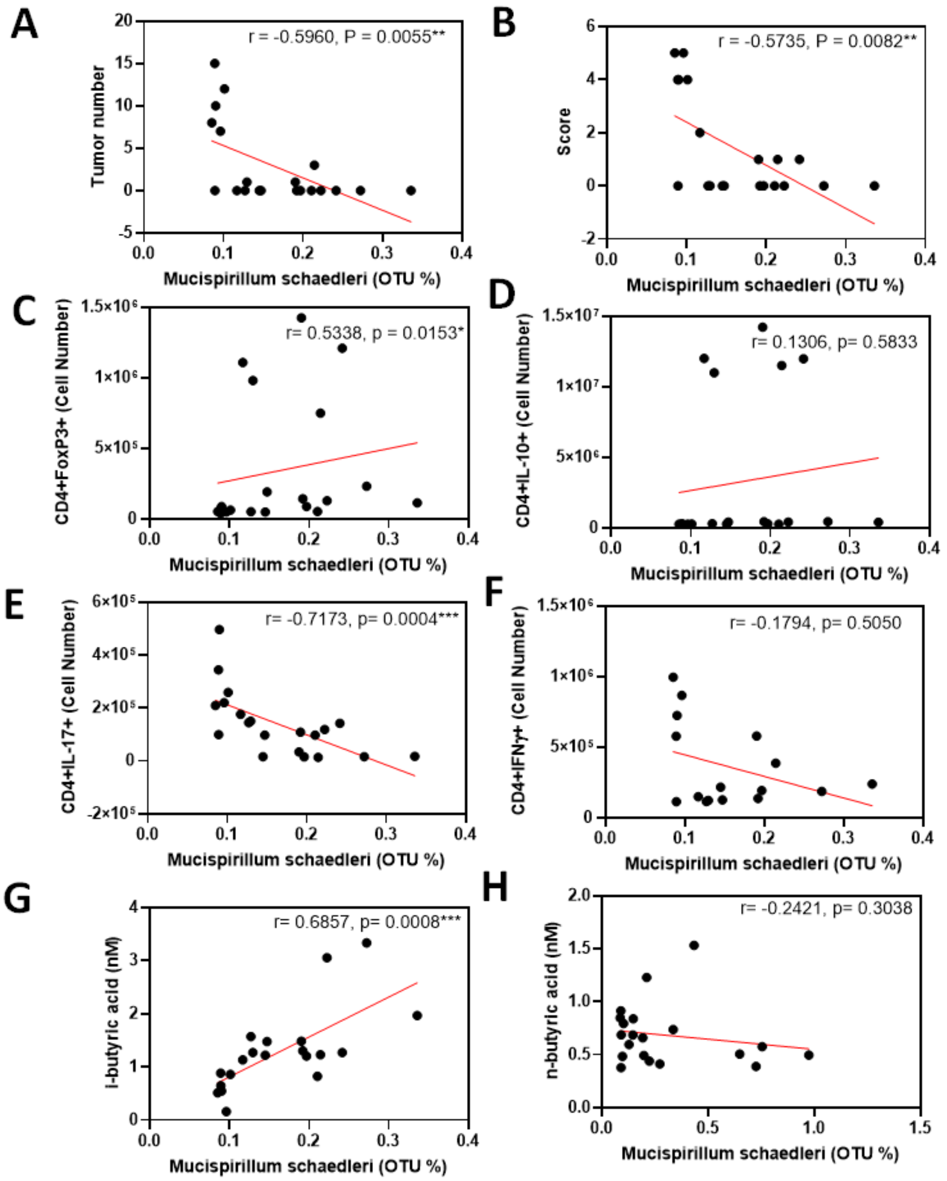

Figure S10. Correlation between Mucispirillum schaedleri abundance and clinical parameters. Spearman correlations were performed between OTU percentages of Mucispirillum schaedleri from experimental groups (Naïve, Resveratrol, AOM, AOM+Reservatrol; n=5 per group) to other variables to include (**a**) tumor number, (**b**) disease score, (**c-f**) Th subsets, and (**g-h**) i-/n-butyric concentrations. Linear regression line (red), rank coefficient (r), and p-values (p) are depicted in the boxes of each comparison. Significance was determined as follows: p-value: *<0.05, **<0.01, ***<0.005, ****<0.001.

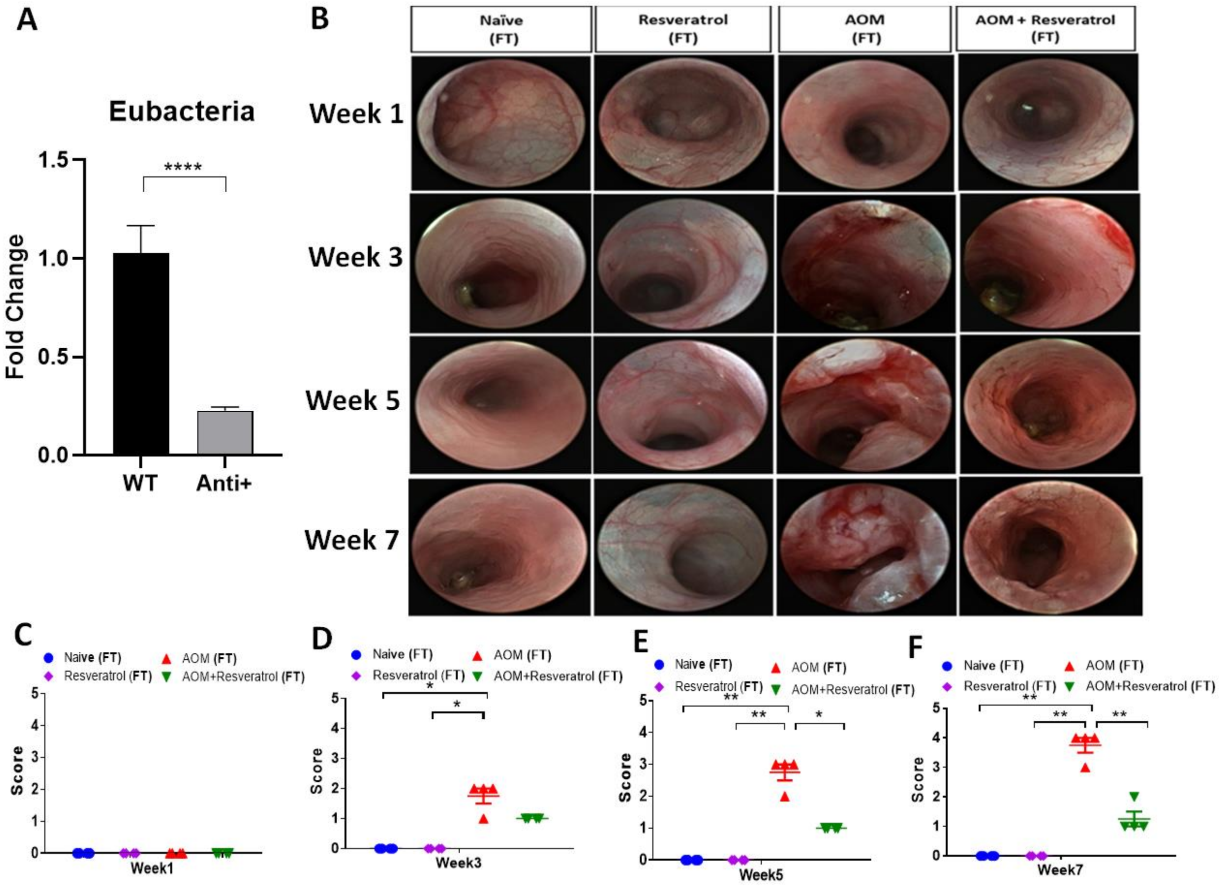

Figure S11: Validation and weekly colonoscopy images in FT experiments. Induction of AOM CRC and FT were performed as described in Figure 3 legend.(a) PCR validation showing expression of Eubacteria from wild-type (WT) control mice (n=4) and mice treated with antibiotics (anti+, n=16). (**b**) Representative colonoscopies are shown from experimental groups which included Naïve(FT) (n=4), Resveratrol(FT) (n=4), AOM(FT) (n=4), AOM+Resveratrol(FT) (n=4) at weeks 1, 3, 5, and 7. Bar graphs depict colonoscopy scores (described in Materials and Methods) for experimental groups at week 1 (**c**), week 3 (**d**), week 5 (**e**), and week 7 (**f**). Significance (p-value: *<0.05, **<0.01, ***<0.005, ****<0.001) was determined by using one-way ANOVA followed by Tukey’s post-hoc multiple comparisons test for depicted bar graphs and student’s t test was used in the PCR validation.

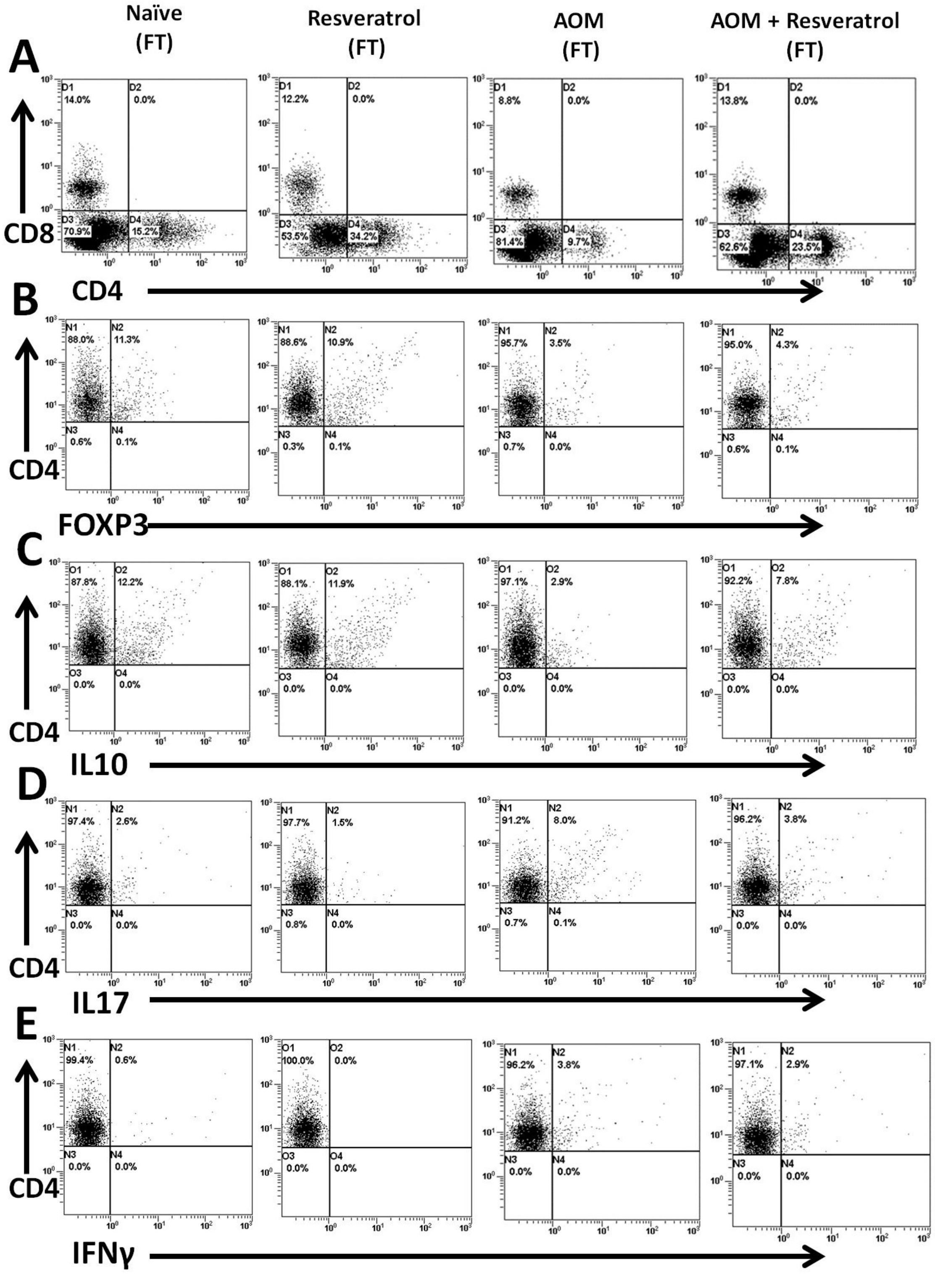

Figure S12. T cell phenotyping in MLN of FT experiments. Induction of AOM CRC and FT were performed as described in Figure 3 legend. Representative flow cytometry dot plots are depicted for the following T cell subsets: CD3+CD4+CD8+ (**a**), CD4+FOXP3+ (**b**), CD4+IL10+ (**c**) and CD4+IFNγ+ (**d**), and CD4+IL-17+ (**e**). For b-e, cells were gated on CD4+ population. Data are representative of at least 3 independent experiments.

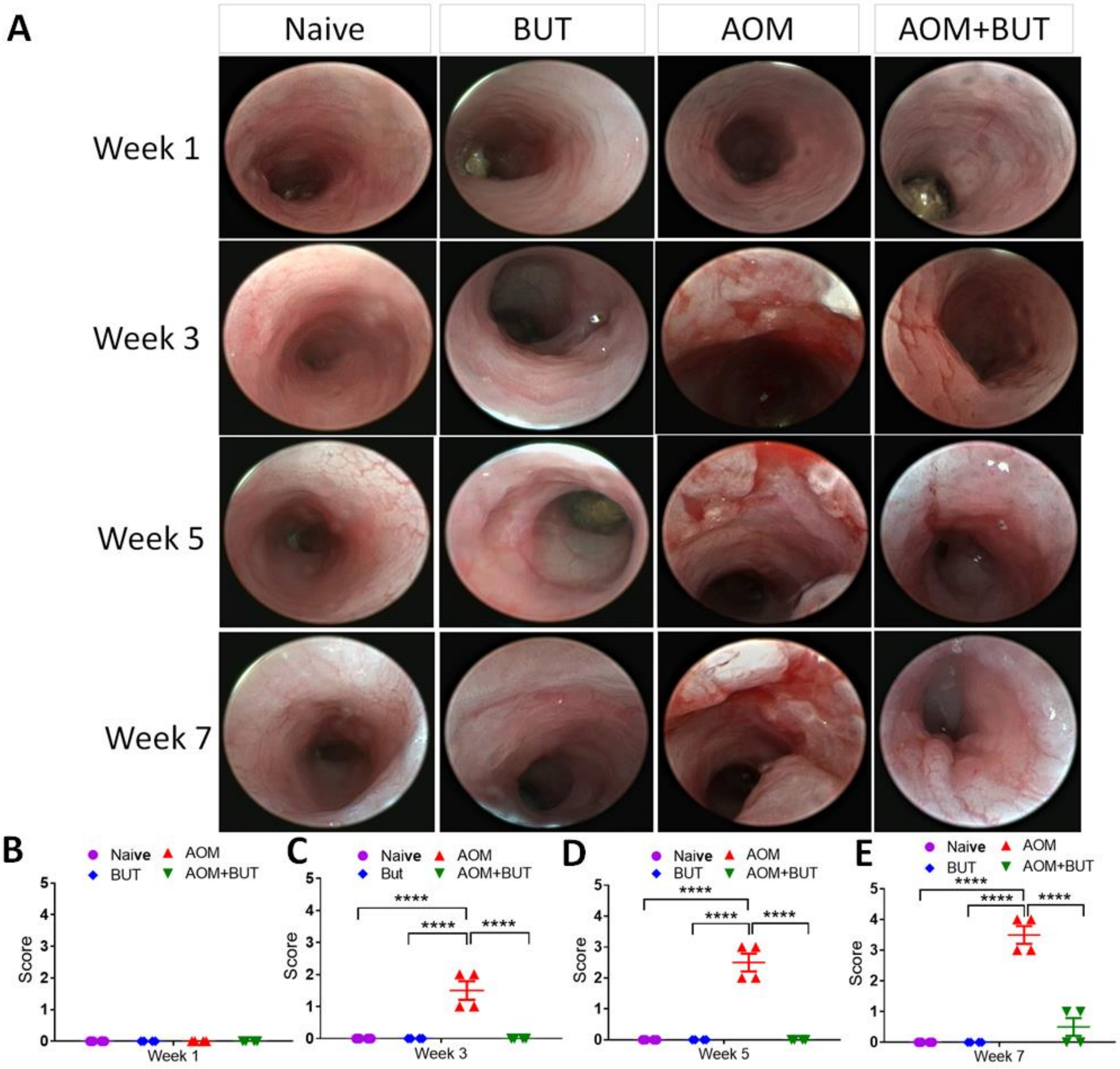

Figure S13. Weekly colonoscopy images in AOM-induced CRC treated with BUT. Induction of AOM CRC and treatment with resveratrol was performed as described in Figure 4 legend. (**a**) Representative colonoscopies are shown from experimental groups which included Naïve (n=4), BUT (n=4), AOM (n=4), AOM+BUT (n=4) at weeks 1, 3, 5, and 7. Bar graphs depict colonoscopy scores (described in Materials and Methods) for experimental groups at week 1 (**b**), week 3 (**c**), week 5 (**d**), and week 7 (**e**). Significance (p-value: *<0.05, **<0.01, ***<0.005, ****<0.001) was determined by using one-way ANOVA followed by Tukey's post-hoc multiple comparisons test for depicted bar graphs.

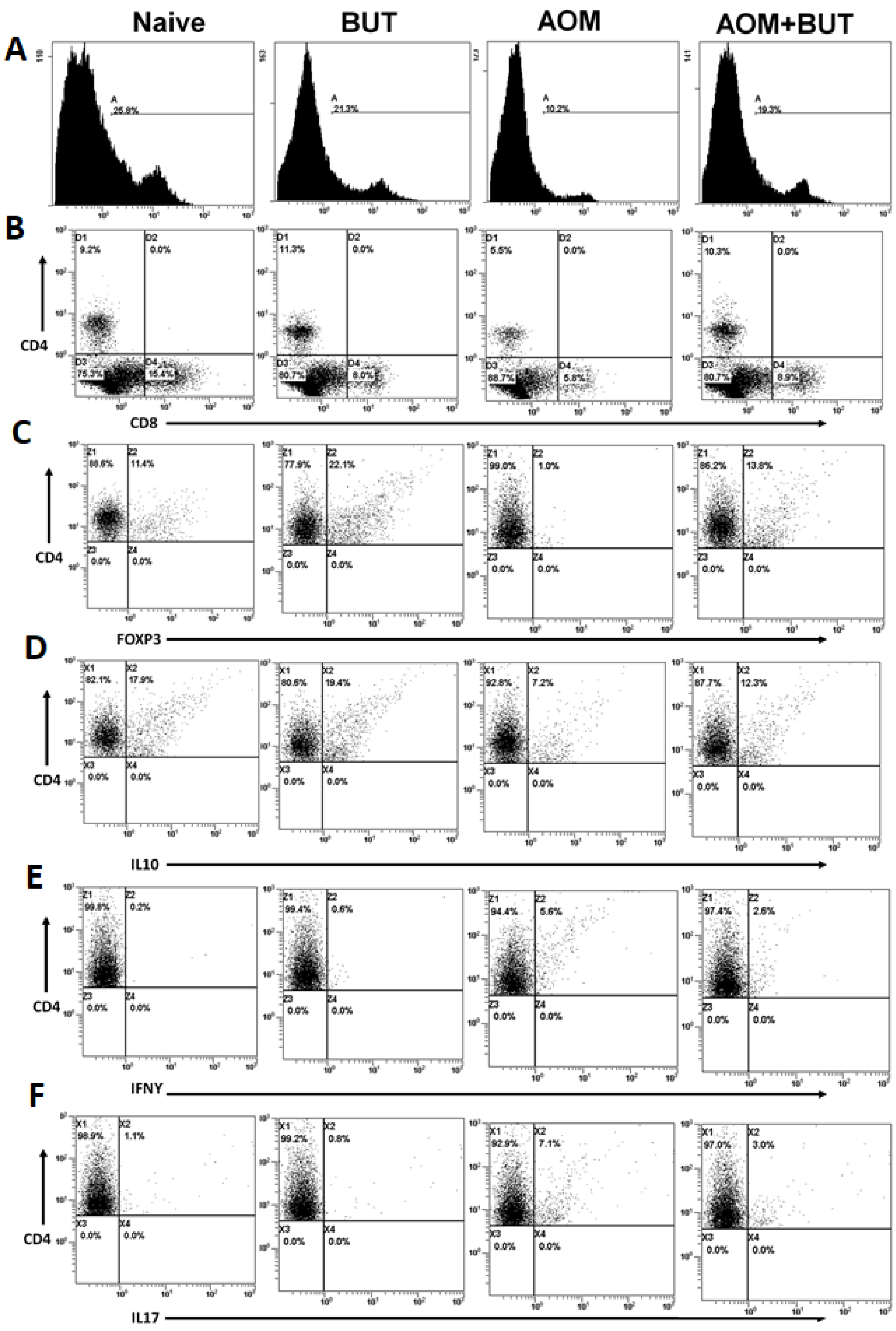

Figure S14. T cell phenotyping in MLN of AOM-induced CRC mice treated with BUT. Induction of AOM CRC and treatment with resveratrol was performed as described in Figure 4 legend. Representative flow cytometry histograms and dot plots are depicted for the following T cell subsets: CD3+ (**a**), CD3+CD4+CD8+ (**b**), CD4+FOXP3+ (**c**), CD4+IL10+ (**d**) and CD4+IFNγ+ (**e**), and CD4+IL-17+ (**f**). For Figures c-f, cells were gated on CD4+ population. Data are representative of at least 3 independent experiments.

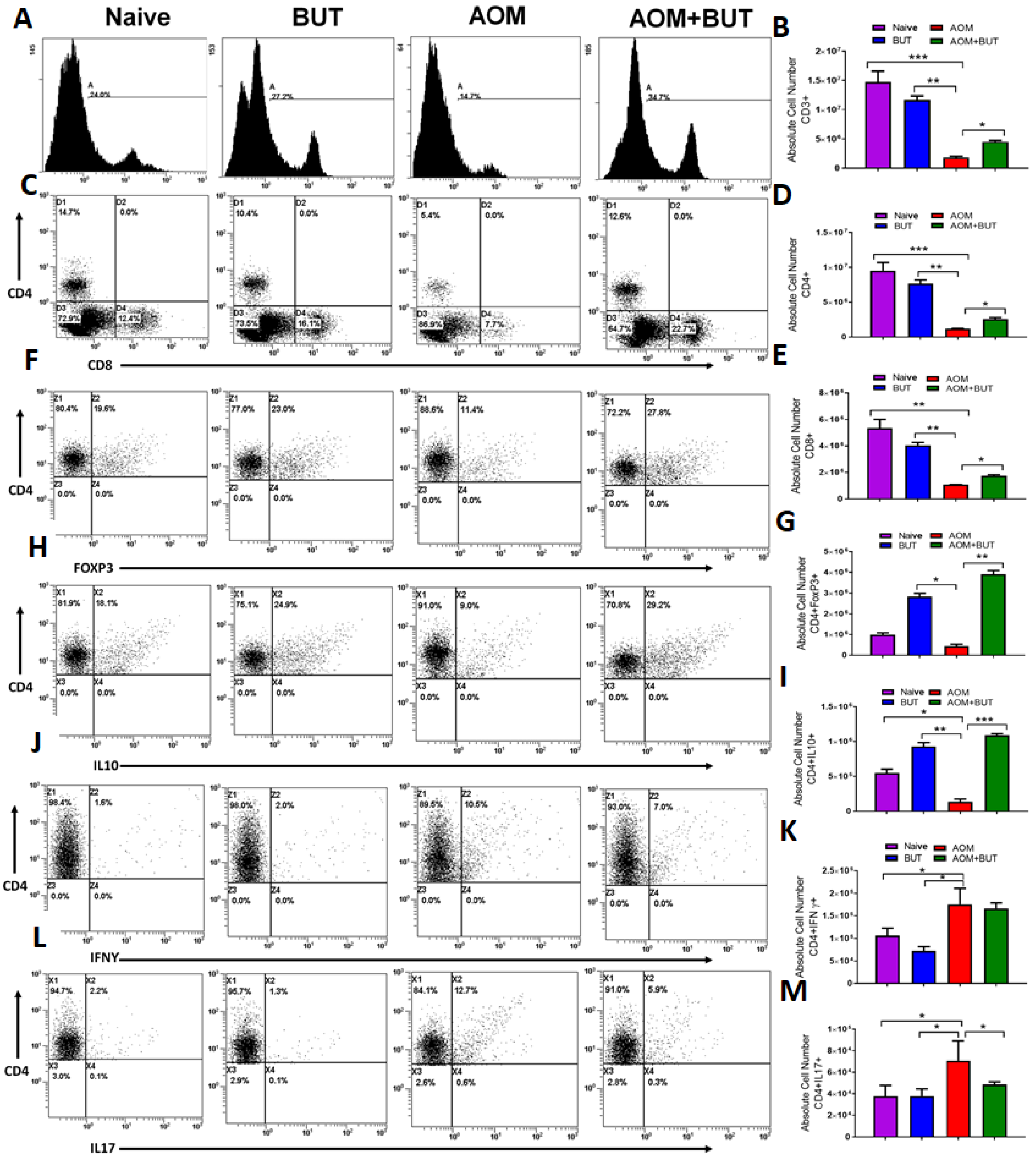

Figure S15. T cell phenotyping in spleen of AOM-induced CRC mice treated with BUT. Induction of AOM CRC and treatment with resveratrol was performed as described in Figure 4 legend. Flow cytometry dot plots and quantitative bar graphs depicting absolute cell numbers are shown respectively for the following T cell subsets: CD3+ (**a-b**), CD4+ or CD8+ cells (**c-e**), CD4+FOXP3+ (**f-g**), CD4+IL10+ (**h-i**) and CD4+IFNγ+ (**j-k**), and CD4+IL-17+ (**l-m**) expressing cells. For Figures f-m, cells were gated on CD4+ population. Each experimental group had at least 5 mice included, and significance (p-value: *<0.05, **<0.01, ***<0.005, ****<0.001) was determined for absolute cell numbers by using one-way ANOVA followed by Tukey's post-hoc multiple comparisons test. Data are representative of at least 3 independent experiments.

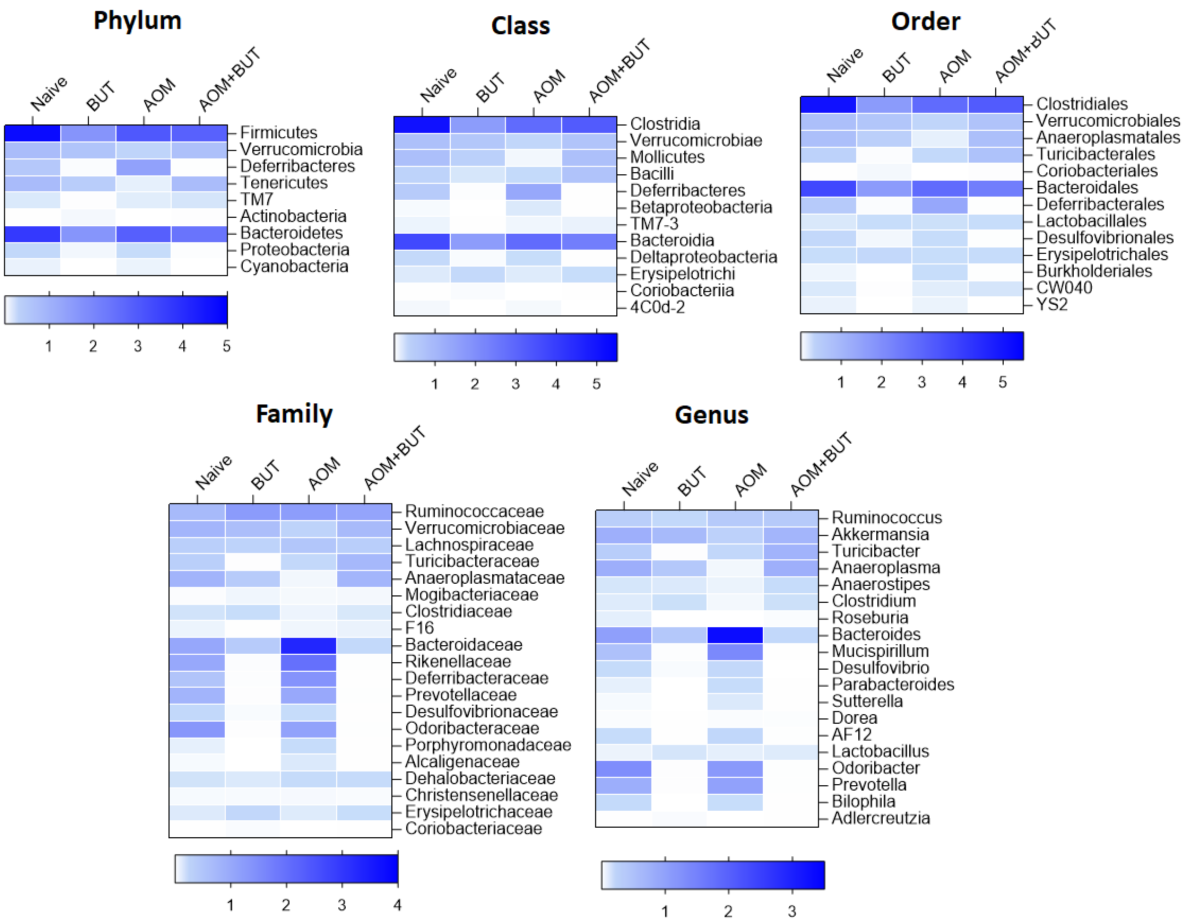

Figure S16. Significantly altered bacteria in AOM-induced CRC sample treated with BUT at the phylum to genus levels. Induction of AOM CRC and treatment with resveratrol was performed as described in Figure 4 legend, and 16S rRNA sequencing was performed as described in Figure 5 legend. Bacteria depicted in rows were found to be significantly different (p-value: <0.05) between one or more of the experimental groups using one-way ANOVA and Tukey's multiple comparisons test. Columns represent the experimental groups: : Naïve (n=5), BUT (n=5), AOM (n=5), and AOM+BUT (n=5), and each cell represents the mean OTU abundance of the corresponding bacteria with SEM.

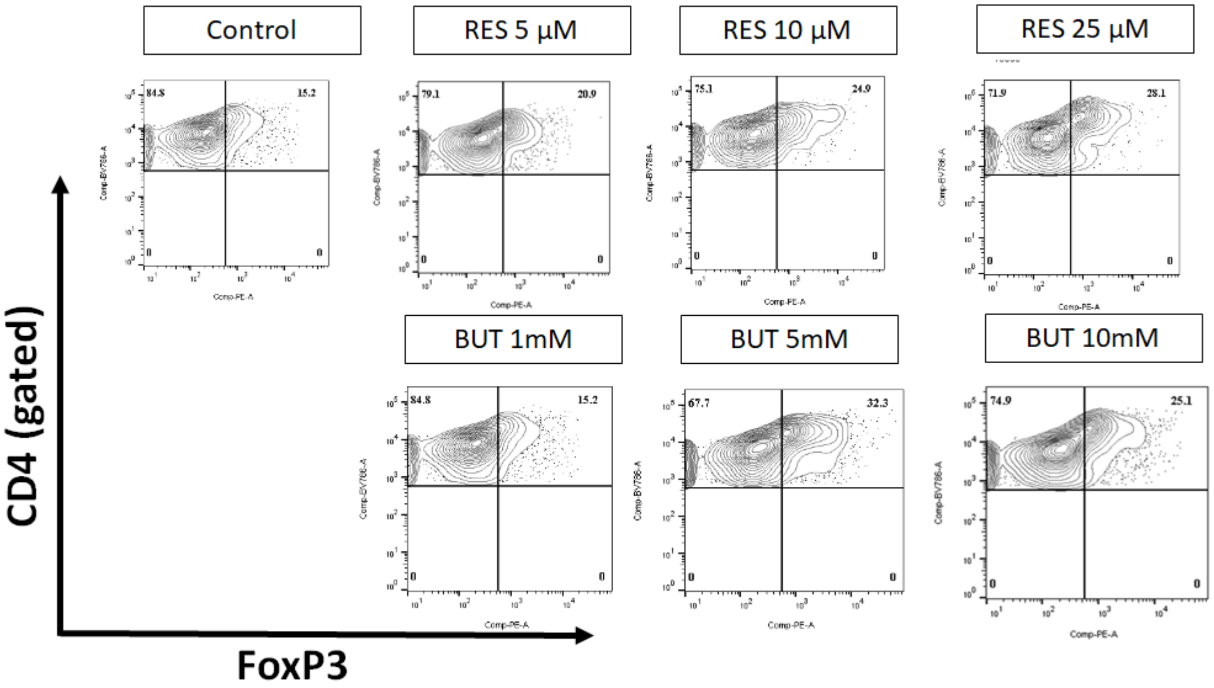

Figure S17. Resveratrol and BUT dose-dependently increase Tregs *in vitro*. Whole splenocytes (seeded at 1 x 10^6 cells/ml) from 8-10 week old C57BL/6 mice were activated using CD3 (.5μg/ml) and CD28 (2μg/ml) in the absence or presence of appropriate vehicle control, resveratrol (5, 10, or 25μM), or BUT (1, 5, or 10mM). After 24 hours, cells were fixed and stained with antibodies to identify percentages of Tregs (CD4+FoxP3+). Plots are representative flow dot plots for vehicle control and the various doses of RES and BUT gated on the CD4 population. A total of 3 independent wells (n=3) were used for each group and the data is representative of 2 independent experiments.

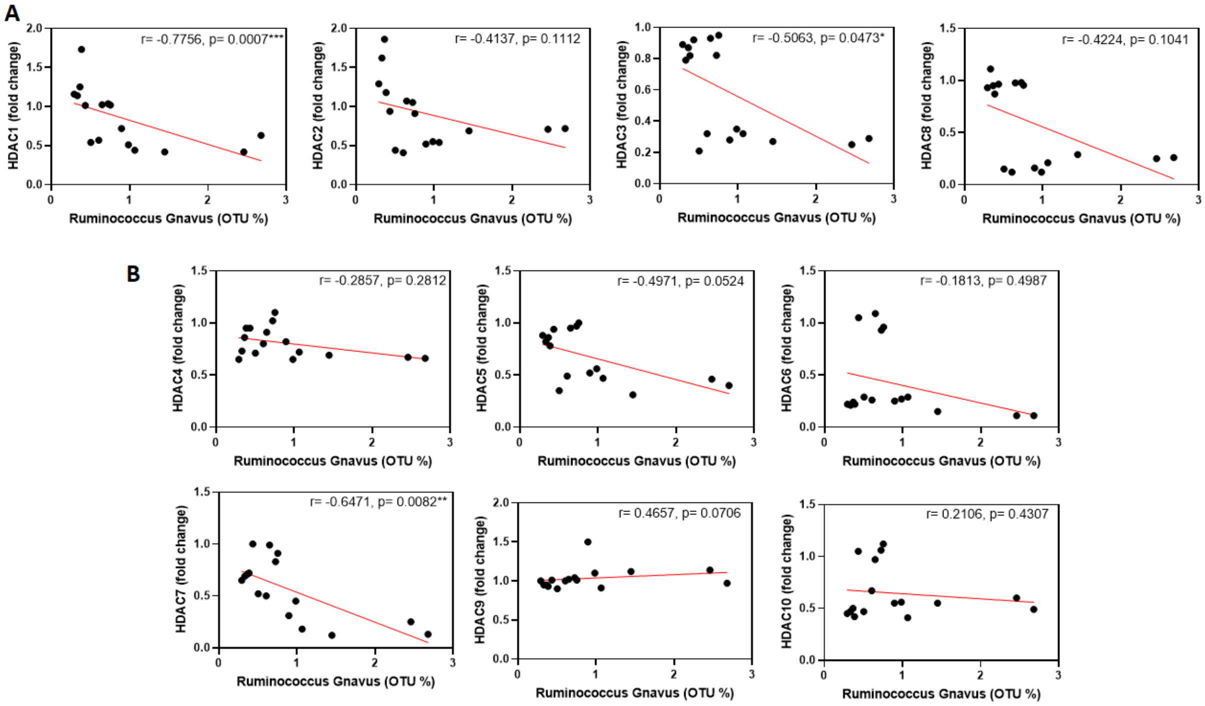

Figure S18: Correlation between Ruminococcus gnavus abundance and HDAC expression. Spearman correlations were performed between OTU percentages of Ruminococcus gnavus from experimental groups (Naïve, Resveratrol, AOM, AOM+Reservatrol; n=5 per group) to HDACs to include (**a**) Class I, and, (**b**) Class II HDACs. Linear regression line (red), rank coefficient (r), and p-values (p) are depicted in the boxes of each comparison. Significance was determined as follows: p-value: *<0.05, **<0.01, ***<0.005, ****<0.001.

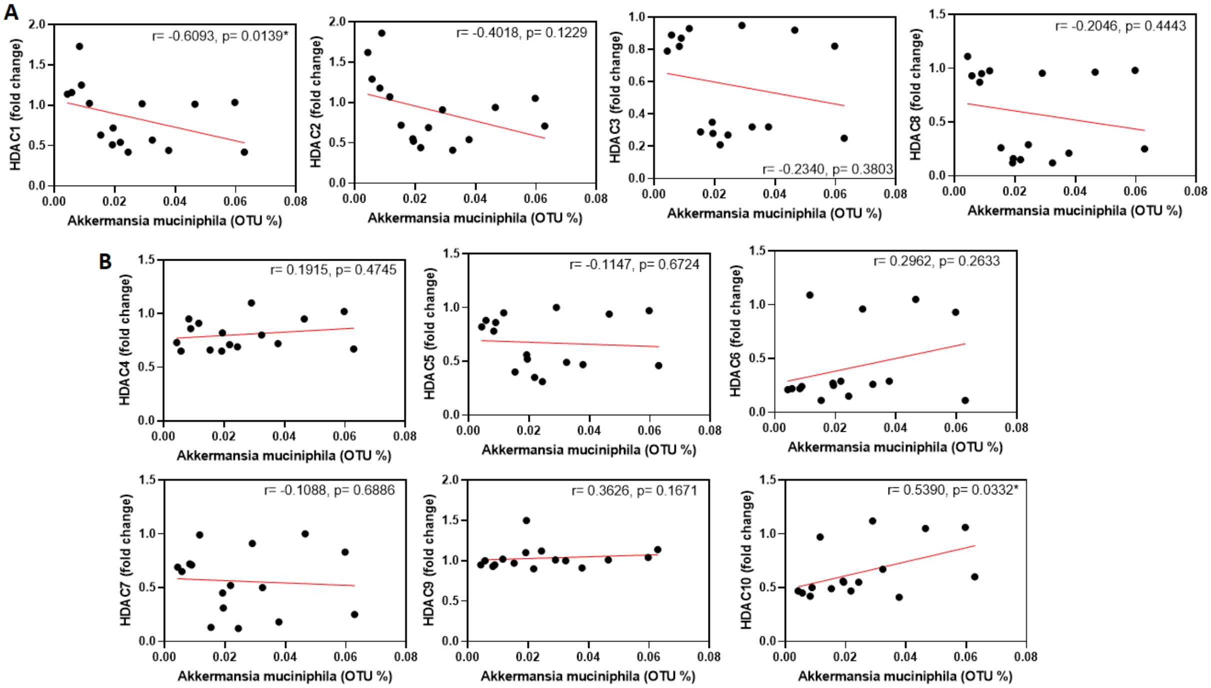

Figure S19: Correlation between Akkermansia muciniphilia abundance and HDAC expression. Spearman correlations were performed between OTU percentages of Akkermansia muciniphilia from experimental groups (Naïve, Resveratrol, AOM, AOM+Reservatrol; n=5 per group) to HDACs to include (**a**) Class I, and, (**b**) Class II HDACs. Linear regression line (red), rank coefficient (r), and p-values (p) are depicted in the boxes of each comparison. Significance was determined as follows: p-value: *<0.05, **<0.01, ***<0.005, ****<0.001.

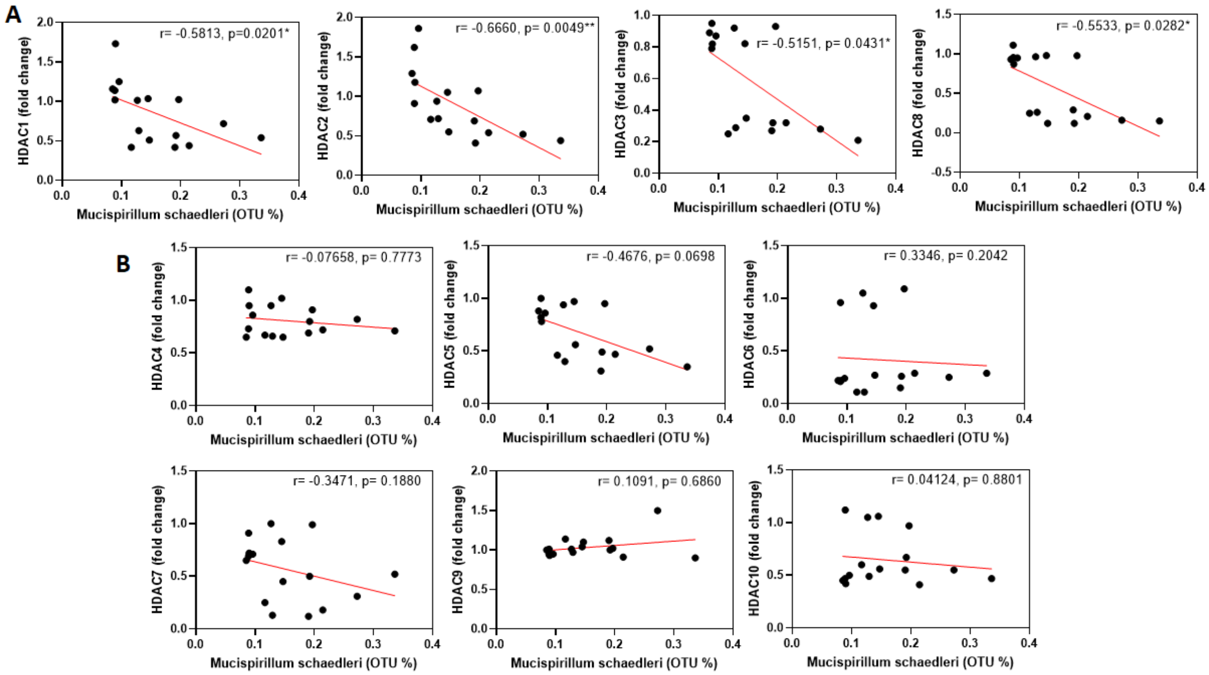

Figure S20: Correlation between Mucispirillum schaedleri abundance and HDAC expression. Spearman correlations were performed between OTU percentages of Mucispirillum schaedleri from experimental groups (Naïve, Resveratrol, AOM, AOM+Reservatrol; n=5 per group) to HDACs to include (**a**) Class I, and, (**b**) Class II HDACs. Linear regression line (red), rank coefficient (r), and p-values (p) are depicted in the boxes of each comparison. Significance was determined as follows: p-value: *<0.05, **<0.01, ***<0.005, ****<0.001.
